# Hierarchical Chemical Fluctuations Promote Exceptional Strength–Ductility–Modulus Synergy in Biomedical BCC Multi‐Principal‐Element Alloys

**DOI:** 10.1002/advs.78661

**Published:** 2026-11-09

**Authors:** Jiakun Wang, Jiayao Zhong, Jinxi Guo, Jonathan Aristya, Minghang Zhou, Liang Zhu, Xipeng Tan, Jinfu Li

**Affiliations:** ^1^ State Key Laboratory of Metal Matrix Composites School of Materials Science and Engineering Shanghai Jiao Tong University Shanghai China; ^2^ Department of Mechanical Engineering National University of Singapore Singapore Singapore

**Keywords:** biomedical multi‐principal‐element alloys, dislocation interactions, hierarchical chemical fluctuations, kink bands, strength‐ductility‐modulus synergy

## Abstract

Simultaneously achieving high strength, large tensile ductility, and low Young's modulus remains a central challenge in body‐centered‐cubic (BCC) multi‐principal‐element alloys (MPEAs) to be used as load‐bearing biomedical materials. Here, we show a strategy of combining thermodynamic considerations and valence electron theory to address this issue. By means of the contrasting interactions of Sn with group‐IV and group‐V elements, hierarchical chemical fluctuations (CFs) spanning from <1 to ∼50 nm and corresponding alternating strain fields were introduced in the optimized Ti_35_Zr_35_Nb_10_Hf_10_Ta_5_Sn_5_ alloy, favoring dislocation pinning, pile‐up, and junction formation, thereby facilitating kink band formation early during uniform deformation and subsequent kink‐band‐mediated plasticity. Consequently, the as‐cast alloy achieves a yield strength of ∼1.14 GPa, tensile elongation of 20.3%, and Young's modulus of 52.8 GPa, together with improved cytocompatibility relative to the Sn‐free parent alloy. Kink bands represent a special plastic deformation mode, as their formation can accommodate localized strains, alleviate stress concentration, and avoid premature fracture. This work not only provides a novel pathway for realizing excellent strength–ductility–modulus synergy in BCC MPEAs, but also brings insight into the action of kink bands in partitioning plastic deformation that has not been well understood before.

## Graphical abstract

Hierarchical chemical fluctuations are engineered in a biomedical BCC Ti_35_Zr_35_Nb_10_Hf_10_Ta_5_Sn_5_ multi‐principal‐element alloy through contrasting Sn–element interactions. The resulting multiscale chemical–strain heterogeneity promotes dislocation interactions and kink‐band‐mediated partitioned plasticity, enabling the as‐cast alloy to achieve a yield strength of 1.14 GPa, a tensile elongation of 20.3%, and a Young's modulus of 52.8 GPa.

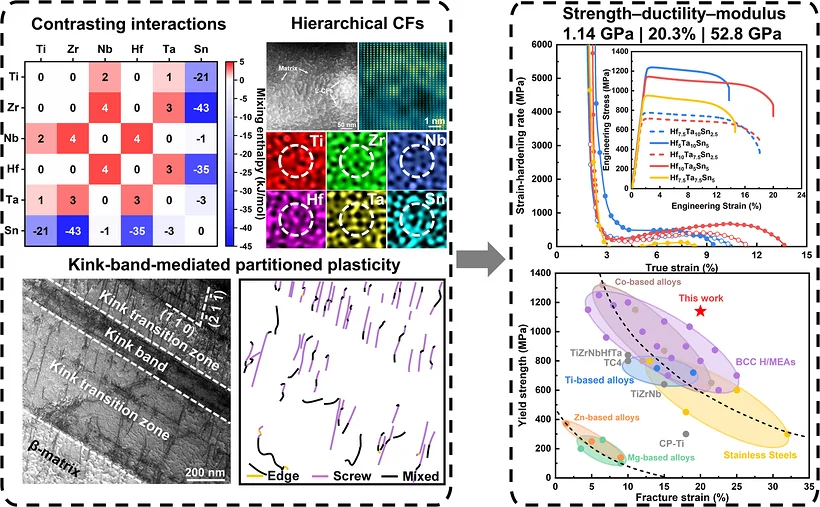

## 1 Introduction

The strength–ductility trade‐off remains a central challenge in metallic materials, particularly in body‐centered‐cubic (BCC) multi‐principal‐element alloys (MPEAs) [1, 2, 3, 4]. Their room‐temperature deformation is usually characterized by localized dislocation glide, limited strain hardening, and premature strain localization [5, 6, 7, 8]. Various strategies, including metastable phase engineering [9], interstitial‐mediated strengthening [10, 11], heterogeneous microstructure design [12, 13], and precipitation strengthening [14, 15], have been proposed to improve the strength–ductility balance. However, many of these strategies require additional processing steps or carefully controlled microstructural states, making robust strength–ductility synergy difficult to achieve in as‐cast BCC alloys. The challenge becomes more stringent for the alloys to be used as load‐bearing biomedical materials where a low Young's modulus and good biocompatibility are additionally required to mitigate stress shielding and promote bone regeneration [16, 17].

Plastic deformation primarily relies on dislocation multiplication and motion, and in some cases on phase transformation or twinning. Beyond these mechanisms, kink bands provide a potential route to coordinate plastic deformation in BCC alloys [18, 19, 20]. By accommodating local strain through lattice rotation and dislocation rearrangement, kink bands can alleviate stress concentration and delay fracture [21, 22, 23]. In many reported BCC systems, however, kink bands are introduced or enhanced by cold working, thermomechanical processing, or heat treatment, making their formation strongly dependent on processing history [24, 25, 26]. Mechanistically, kink bands have been interpreted as geometrical instabilities associated with heterogeneous slip and lattice rotation [26], twinning‐like rotational domains formed by cooperative dislocation motion [27], or deformation structures that develop when available slip systems become exhausted [28]. Some studies have related kink‐band formation to specific slip‐system selection or local stress states, including low‐valence‐electron‐concentration group‐IV alloying that promotes {112}<111> slip [22], and the combined effects of necking, hard particles, and slip exhaustion [29]. These descriptions clarify important crystallographic characteristics and specific deformation conditions associated with kink‐band formation, but they rarely consider the role of local chemical environments, even though chemical heterogeneity and internal stress fluctuations are known to strongly influence defect motion and strength–ductility synergy in MPEAs [30, 31, 32]. A key unresolved question is therefore whether kink‐band‐mediated plasticity can be actuated by tailoring local chemical heterogeneity in a β‐BCC matrix.

Ti–Zr–Nb–Hf–Ta‐based β‐BCC MPEAs provide a useful platform for addressing this question. This alloy family can access a single β‐BCC matrix and contains constituent elements with potential biomedical relevance [33, 34, 35], exhibiting promising combinations of strength, ductility, and elastic modulus [36, 37, 38, 39]. In our previous study, an as‐cast Ti_35_Zr_35_Nb_10_Hf_10_Ta_10_ alloy, hereafter denoted as Hf_10_Ta_10_, showed an attractive strength–ductility–modulus balance, and Hf/Ta‐related local chemical fluctuations were correlated with kink‐band‐mediated plastic deformation [40]. However, kink bands in Hf_10_Ta_10_ were mainly observed at high strains, particularly near necking or after fracture, as commonly reported in other BCC alloys [20, 23, 29]. This raises two further issues: can kink bands be introduced at an early stage of uniform plastic flow, and what measures can effectively realize this purpose?

In this work, we explored the role of Sn in modulating the Hf_10_Ta_10_ β‐BCC alloy. Sn was selected because it combines low elastic modulus, proper biocompatibility, and pronounced atomic‐size/modulus mismatch with the Ti–Zr–Nb–Hf–Ta matrix [41, 42, 43]. More importantly, Sn provides a marked chemical‐affinity contrast within the alloy, as reflected by strongly negative mixing enthalpies with the group‐IV elements Ti, Zr, and Hf but much weaker interactions with the group‐V elements Nb and Ta [44]. This coupled chemical–misfit perturbation provides a thermodynamic and structural basis for enhancing chemical fluctuations within a retained single β‐BCC framework. Our experiments show that the addition of Sn indeed generates hierarchical chemical fluctuations (CFs) in the optimized Ti_35_Zr_35_Nb_10_Hf_10_Ta_5_Sn_5_ alloy (hereafter denoted as Hf_10_Ta_5_Sn_5_). These CFs, accompanied by local lattice‐strain heterogeneity, favor dislocation pinning, pile‐up, tangling, and junction formation, thereby facilitating kink‐band‐related lattice rotation early during uniform plastic deformation. Together with metastable‐β low‐modulus design, these coupled effects enable the as‐cast Hf_10_Ta_5_Sn_5_ alloy, without additional thermomechanical processing, to achieve a high yield strength of ∼1.14 GPa, a large fracture elongation of 20.3%, and a low Young's modulus of 52.8 GPa, besides improving biocompatibility. This property combination suggests high relevance to load‐bearing biomedical implant materials, while the underlying mechanism establishes local chemical heterogeneity as a design variable for guiding kink‐band‐mediated plasticity in BCC MPEAs.

## 2 Results and Discussion

### 2.1 Alloy Design and Tensile Properties

A series of Sn‐containing alloys, Ti_35_Zr_35_Nb_10_Hf_7.5_Ta_10_Sn_2.5_, Ti_35_Zr_35_Nb_10_Hf_5_Ta_10_Sn_5_, Ti_35_Zr_35_Nb_10_Hf_10_Ta_7.5_Sn_2.5_, Ti_35_Zr_35_Nb_10_ Hf_10_Ta_5_Sn_5_ and Ti_35_Zr_35_Nb_10_Hf_7.5_Ta_7.5_Sn_5_ (in at.%), were designed by partial substitution of Hf and/or Ta by Sn in the Ti_35_Zr_35_Nb_10_Hf_10_Ta_10_ parent alloy. This substitution strategy was intended to retain a β‐BCC‐compatible phase window while perturbing the average electronic structure and local thermodynamic‐interaction landscape of the parent matrix (Note S1). The elemental physical and d‐electron parameters used for the alloy‐design calculations are summarized in Table S1. For brevity, the alloys are denoted as Hf_7.5_Ta_10_Sn_2.5_, Hf_5_Ta_10_Sn_5_, Hf_10_Ta_7.5_Sn_2.5_, Hf_10_Ta_5_Sn_5_, Hf_7.5_Ta_7.5_Sn_5_ and Hf_10_Ta_10_, respectively. These alloys have valence electron concentration (VEC) values in the range of 4.15–4.20 and are located within the metastable‐β‐related region in the $\bar{Bo}$–$\bar{Md}$ diagram (Figure S1 and Table S2). Consistently, electron backscatter diffraction (EBSD) phase maps show that all as‐cast alloys retain a single β‐BCC structure, without detectable secondary phases at the EBSD scale (Figure S2). The corresponding inverse pole figure (IPF) maps reveal equiaxed grains with no pronounced initial texture. Compared with the Sn‐free Hf_10_Ta_10_ parent alloy that has an average grain size of ∼60.9 µm, the Sn‐containing alloys exhibit refined grain sizes of ∼29.8–43.9 µm, indicating that Sn addition preserves the β‐BCC phase while refining the solidification microstructure.

The room‐temperature tensile responses of the Sn‐containing alloys are summarized in Figure 1a. All compositions exhibit continuous plastic flow after yielding, but their strength, ductility, and strain‐hardening behavior depend strongly on the Hf/Ta/Sn ratio. The Hf_5_Ta_10_Sn_5_ alloy shows the highest yield strength of 1238 MPa, but its fracture elongation decreases to 13.8%. By contrast, Hf_10_Ta_5_Sn_5_ achieves a more favorable balance, with a yield strength of 1143 MPa, a fracture elongation of 20.3%, and a Young's modulus of 52.8 GPa (Table S2). Compared with Sn‐free Hf_10_Ta_10_, Hf_10_Ta_5_Sn_5_ shows a marked increase in yield strength while retaining substantial tensile ductility and a similarly low elastic modulus. All compositions show a rapid decrease in strain‐hardening rate after yielding, while Hf_10_Ta_5_Sn_5_ retains a moderate positive hardening response over part of the subsequent uniform deformation range. This behavior is associated with continued dislocation storage and interactions during plastic deformation [43, 45].

**FIGURE 1:**
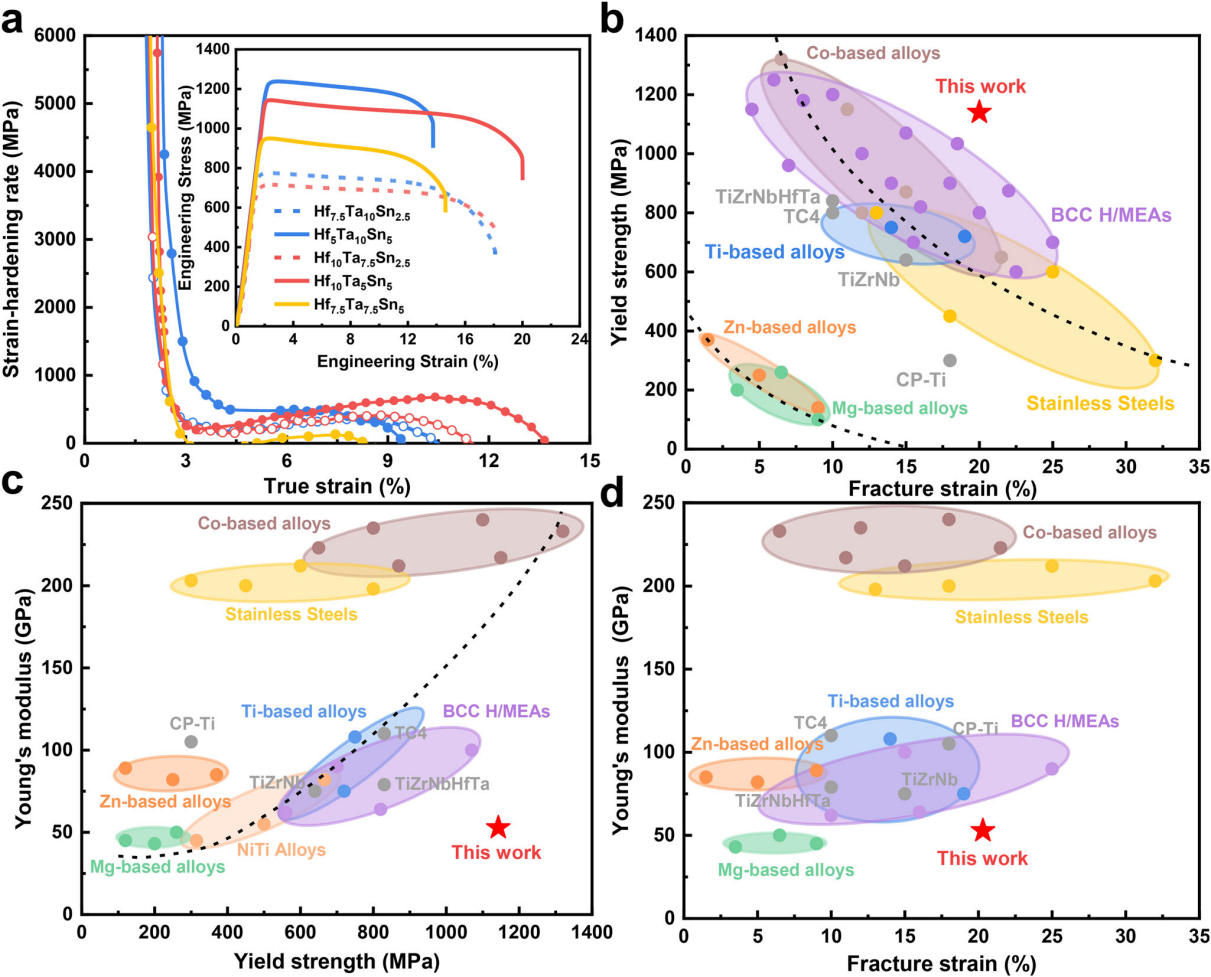
Tensile properties and strength–ductility–modulus benchmark plots of the Sn‐alloyed Ti–Zr–Nb–Hf–Ta alloys. **a**, Strain‐hardening rate as a function of true strain for the investigated Sn‐containing alloys. The inset shows the corresponding engineering stress–strain curves. **b–d**, Property maps comparing the Hf_10_Ta_5_Sn_5_ alloy with representative biomedical metallic materials, BCC high‐/medium‐entropy alloys and related multi‐principal‐element alloys: **b**, yield strength versus fracture strain; **c**, Young's modulus versus yield strength; **d**, Young's modulus versus fracture strain. The Hf_10_Ta_5_Sn_5_ alloy developed in this work is marked by the red star.

The property maps in Figure 1b–d benchmark the Hf_10_Ta_5_Sn_5_ alloy against representative biomedical metallic materials and recently reported BCC high‐/medium‐entropy alloys (H/MEAs) and related MPEAs [7, 33, 34, 36, 45, 46, 47, 48, 49, 50, 51, 52, 53, 54]. Compared with conventional biomedical metals that often suffer from either insufficient strength, limited ductility, or high elastic modulus, and most BCC H/MEAs whose high strength is commonly accompanied by limited tensile plasticity, the as‐cast Hf_10_Ta_5_Sn_5_ alloy occupies a rarely accessed property window. It combines gigapascal‐level yield strength with large tensile ductility while retaining a low Young's modulus, thereby addressing the additional modulus constraint relevant to potential load‐bearing biomedical applications where stress shielding needs to be mitigated [55]. Notably, this combination is achieved in an as‐cast single β‐BCC matrix without additional thermomechanical processing. The fracture surfaces of the alloy series display dimpled morphologies and local tear ridges, confirming ductile microvoid‐coalescence fracture rather than brittle failure (Figure S3). These mechanical and fractographic results demonstrate that an appropriate Hf/Ta/Sn ratio, represented by Hf_10_Ta_5_Sn_5_, enables an unusual strength–ductility–modulus balance. This composition was therefore selected for detailed investigation of the local chemical structure and deformation mechanism.

### 2.2 Hierarchical Chemical Fluctuations and Interaction Contrast

We next examined the local chemical composition and structure of as‐cast Hf_10_Ta_5_Sn_5_ using transmission electron microscopy (TEM) and aberration‐corrected scanning transmission electron microscopy (STEM). A low‐magnification high‐angle annular dark‐field STEM (HAADF‐STEM) image shows numerous curved, ridge‐like bright‐contrast regions embedded in the β matrix (Figure 2a). These features have widths of approximately 10 nm and lengths extending to several tens of nanometers, estimated from the contrast modulations in the HAADF‐STEM images. The corresponding selected‐area electron diffraction (SAED) pattern contains only β‐BCC reflections, indicating that these contrast modulations do not arise from crystallographically distinct precipitates, secondary phases, or ordered structures with a new periodic crystal arrangement. Enlarged HAADF‐STEM imaging and elemental mapping further reveal that the bright regions are compositionally different from the surrounding matrix (Figure S4). Quantitative STEM energy‐dispersive X‐ray spectroscopy (STEM‐EDS) analysis shows that these regions are relatively enriched in Hf, Ta, and Sn compared with the darker β matrix (Table S3). We therefore refer to these long‐wavelength compositionally modulated domains as long‐range chemical fluctuations (L‐CFs).

**FIGURE 2:**
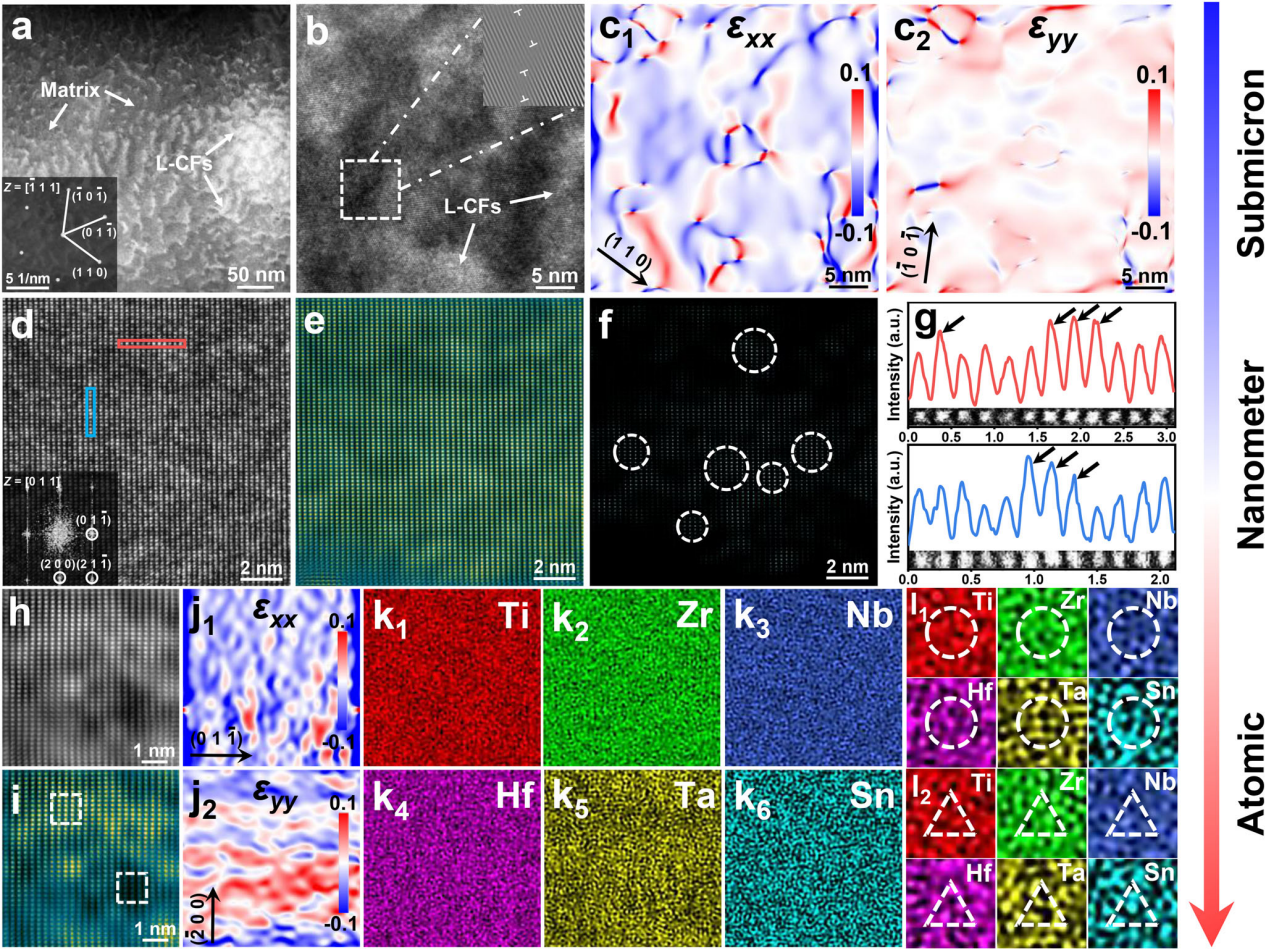
Multiscale TEM/STEM evidence for hierarchical chemical fluctuations in the as‐cast Hf_10_Ta_5_Sn_5_ alloy. **a**, HAADF‐STEM image showing long‐range chemical fluctuations (L‐CFs) embedded in the β matrix, with the corresponding selected‐area electron diffraction pattern shown in the inset. **b**, HRTEM image of an L‐CF‐containing region. **c_1_, c_2_**, Geometric phase analysis strain maps of ε_xx_ and ε_yy_ obtained from **b**, revealing local lattice‐strain fluctuations associated with the chemical fluctuations. **d**, Aberration‐corrected atomic‐resolution HAADF‐STEM image with the corresponding fast Fourier transform pattern shown in the inset. **e**, Inverse fast Fourier transform image reconstructed from **d**, highlighting nanoscale contrast fluctuations. **f**, Extracted contrast‐fluctuation domains in **e; g**, HAADF intensity profiles measured along the marked regions in **d**. **h**, Atomic‐resolution HAADF‐STEM image showing pronounced local contrast variations. **i**, Corresponding inverse fast Fourier transform image showing atomic‐scale contrast fluctuations. **j_1_, j_2_**, GPA strain maps of ε*_xx_* and ε*_yy_* obtained from **h**. **k_1_–k_6_**, STEM‐EDS elemental maps of Ti, Zr, Nb, Hf, Ta and Sn acquired from the region in **h**. **l_1_, l_2_**, Atomic‐scale elemental maps of representative bright‐ and dark‐contrast regions marked in **i**.

The L‐CFs are accompanied by pronounced local lattice‐strain heterogeneity. In an L‐CF‐containing region, high‐resolution TEM imaging reveals a coherent β‐BCC lattice without sharp phase boundaries (Figure 2b). Geometric phase analysis (GPA) maps obtained from this region show alternating tensile and compressive strain fields in both ε*_xx_* and ε*_yy_* (Figure 2c_1_,c_2_). These strain fields are spatially non‐uniform and locally coupled to the contrast‐modulated domains, indicating that the chemical fluctuations are also associated with local lattice distortion rather than being purely compositional features. Such chemical–strain coupling provides a heterogeneous matrix for subsequent dislocation motion.

At the nanometer scale, atomic‐resolution HAADF‐STEM imaging reveals more diffuse contrast modulations within the β matrix (Figure 2d). No additional superlattice spots are observed in the corresponding Fourier‐transform pattern, suggesting that these features are not ordered precipitates with a fixed crystallographic periodicity [56]. Inverse fast Fourier transform reconstruction highlights nanoscale bright and dark domains, and the extracted contrast‐fluctuation map shows that these domains have characteristic dimensions of approximately 1–3 nm (Figure 2e,f). These characteristic dimensions were estimated from the lateral extent of the diffuse bright–dark contrast domains in the reconstructed and contrast‐fluctuation maps. HAADF intensity profiles measured across the marked regions further show repeated intensity oscillations and local intensity spikes (Figure 2g). These nanoscale modulations are defined here as medium‐range chemical fluctuations (M‐CFs).

Chemical heterogeneity and local strain fields further persist down to the atomic‐column scale. High‐resolution HAADF‐STEM images show pronounced bright–dark contrast variations among neighboring atomic columns (Figure 2h,i). GPA strain maps obtained from these regions reveal highly non‐uniform atomic‐scale strain fields (Figure 2j_1_,j_2_), and the corresponding strain profiles show alternating tensile–compressive fluctuations over only a few nanometers (Figure S5). Atomic‐resolution STEM‐EDS maps reveal local compositional variations in Ti, Zr, Nb, Hf, Ta, and Sn (Figure 2k_1_–k_6_). In representative bright‐ and dark‐contrast regions, the elemental maps show atomic‐column‐scale enrichment and depletion of the constituent elements (Figure 2l_1_,l_2_). Elemental line profiles further confirm that the local HAADF intensity variations are accompanied by composition oscillations [36], with heavy elements contributing strongly to the bright contrast while Ti, Zr, and Nb show complementary fluctuations (Figure S6). These sub‐nanometer, atomic‐column‐scale compositional modulations are denoted as short‐range chemical fluctuations (S‐CFs).

These S‐, M‐, and L‐CFs represent chemically correlated fluctuations across different correlation lengths within the same β‐BCC matrix, rather than independent structural domains or ordered phases. Atom probe tomography (APT) provides complementary 3D quantitative analysis for this non‐random chemical landscape. A 5 at.% Sn iso‐concentration surface, chosen to correspond to the nominal Sn content of Hf_10_Ta_5_Sn_5_, reveals spatially heterogeneous Sn distribution within the reconstructed volume (Figure 3a). 1D concentration profiles extracted across representative Sn‐containing regions show coupled fluctuations of the constituent elements rather than sharp compositional plateaus characteristic of well‐defined precipitates (Figure 3b,c). These APT results provide complementary compositional evidence for the Sn‐induced chemical heterogeneity revealed by the multiscale HAADF‐STEM and STEM‐EDS analyses.

**FIGURE 3:**
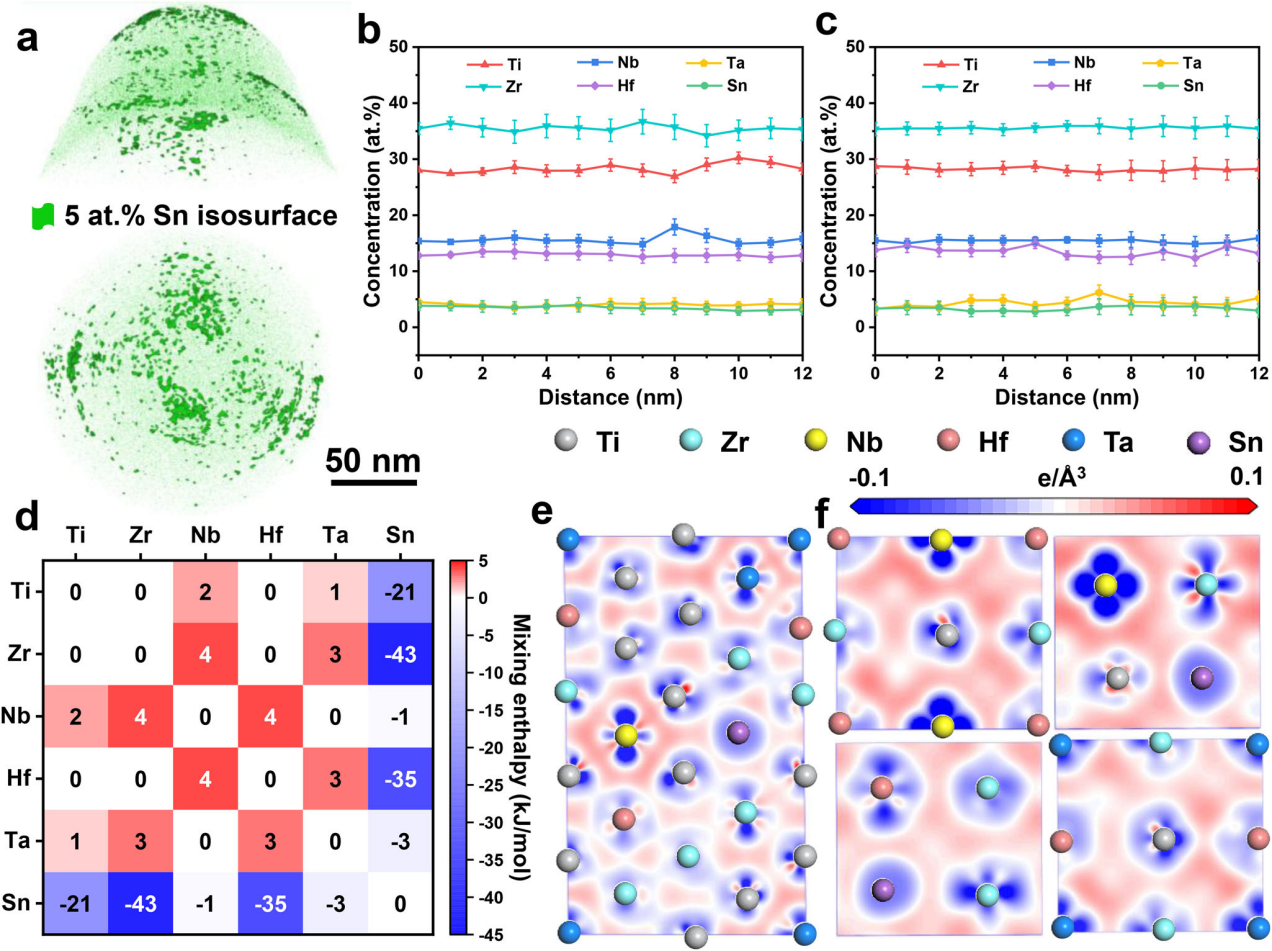
Local chemical heterogeneity and its thermodynamic and electronic origins in the Hf_10_Ta_5_Sn_5_ alloy. **a**, Atom probe tomography reconstruction with a 5 at.% Sn iso‐concentration surface, corresponding to the nominal Sn content and used to visualize spatial heterogeneity in the Sn distribution. **b, c**, 1D concentration profiles extracted across selected Sn‐containing regions, showing coupled local fluctuations of the constituent elements. **d**, Binary mixing enthalpies between the constituent elements, highlighting the contrast between strongly negative Sn–Ti/Zr/Hf interactions and weak Sn–Nb/Ta interactions. **e, f**, DFT‐calculated charge‐density difference maps on representative crystallographic slices, revealing local charge redistribution around different atomic configurations. Red and blue regions denote electron accumulation and depletion, respectively.

The origin of this hierarchical chemical heterogeneity can be understood from the pairwise interaction contrast introduced by Sn. The binary mixing‐enthalpy matrix reveals pronounced contrasts in pairwise interactions, particularly between Sn–group‐IV and Sn–group‐V elemental pairs (Figure 3d). Sn exhibits markedly negative mixing enthalpies with the group‐IV elements Ti, Zr and Hf (−21, −43 and −35 kJ·mol^−1^, respectively), whereas its interactions with the group‐V elements Nb and Ta are much weaker, being close to neutral or only weakly negative (−1 and −3 kJ·mol^−1^, respectively). This interaction contrast provides a thermodynamic bias for non‐random local coordination after Sn incorporation, rather than a fully random distribution of all constituent elements within the β‐BCC solid solution.

Density functional theory (DFT) calculations further show that Sn perturbs the local electronic environment. The charge‐density difference maps reveal spatially heterogeneous electron accumulation and depletion around different local atomic configurations (Figure 3e,f). In Sn‐containing environments, charge redistribution is concentrated near Sn and neighboring Ti/Zr/Hf atoms, indicating locally polarized bonding and a deviation from spatially uniform metallic bonding. Such electronic perturbations are consistent with the pairwise mixing‐enthalpy contrast and with the lattice‐strain heterogeneity detected by GPA in Figure 2. Additional charge‐density difference maps and slice positions are shown in Figure S7, and further discussion of the thermodynamic and electronic origin of the hierarchical chemical fluctuations is provided in Note S2.

### 2.3 Kink‐Band‐Mediated Plastic Deformation

To clarify how the chemically heterogeneous β‐BCC matrix accommodates tensile strain, we combined EBSD‐based orientation analysis with stage‐resolved TEM/STEM observations to characterize the microstructure of Hf_10_Ta_5_Sn_5_ deformed to different stages (Figures 4 and 5; Figure S8). At the yielding stage (∼2% engineering strain), the alloy retains equiaxed β grains (Figure S8a–d). Weak intragranular orientation gradients, together with localized grain reference orientation deviation (GROD) and geometrically necessary dislocation (GND) contrasts, indicate the onset of heterogeneous plastic deformation. TEM observations at this stage reveal localized dislocation clusters, short dislocation segments, cross‐slip traces, and narrow dislocation channels within the β matrix (Figure 5a,b). These features show that plastic flow starts through spatially confined dislocation activity rather than homogeneous slip, establishing an early defect‐heterogeneity state for subsequent orientation evolution.

**FIGURE 4:**
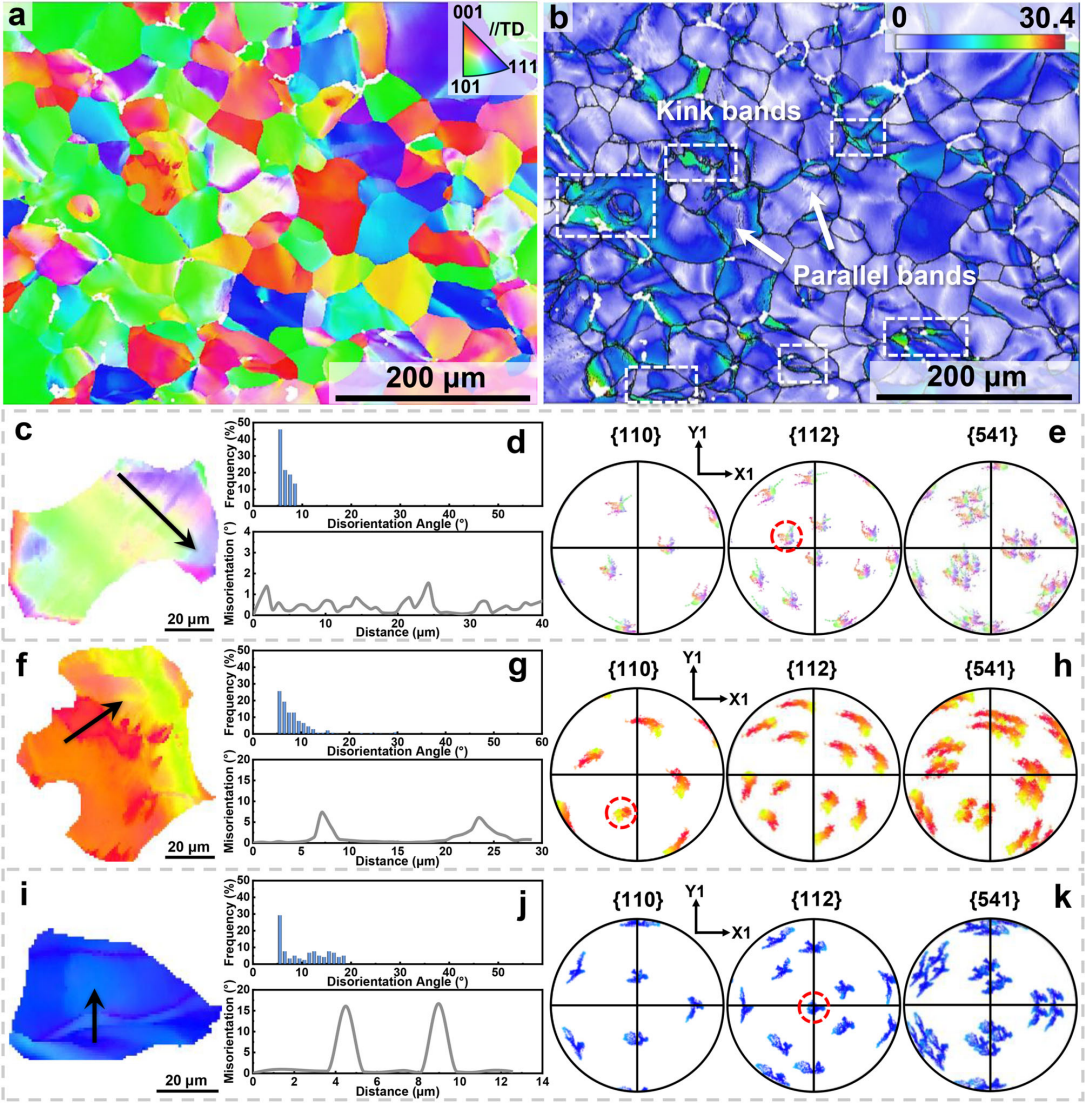
Kink‐band formation in the Hf_10_Ta_5_Sn_5_ alloy during uniform deformation. **a, b**, Inverse pole figure (IPF) and grain reference orientation deviation (GROD) maps showing pronounced intragranular orientation gradients and band‐like deformation structures. TD denotes the tensile direction. **c, f, i**, Representative banded regions selected from **b**, showing kink‐band‐related structures at different degrees of development. The region in **c** contains low‐angle parallel bands with small orientation deviations, whereas the regions in **f** and **i** show more localized orientation changes associated with developed kink bands. **d, g, j**, Disorientation‐angle distributions and point‐to‐point misorientation profiles measured along the arrows in **c**, **f** and **i**, respectively. **e, h, k**, Corresponding {110}, {112} and {541} pole figures, showing pole clustering mainly around <110> or <112> axes and supporting kink‐band formation during uniform deformation.

**FIGURE 5:**
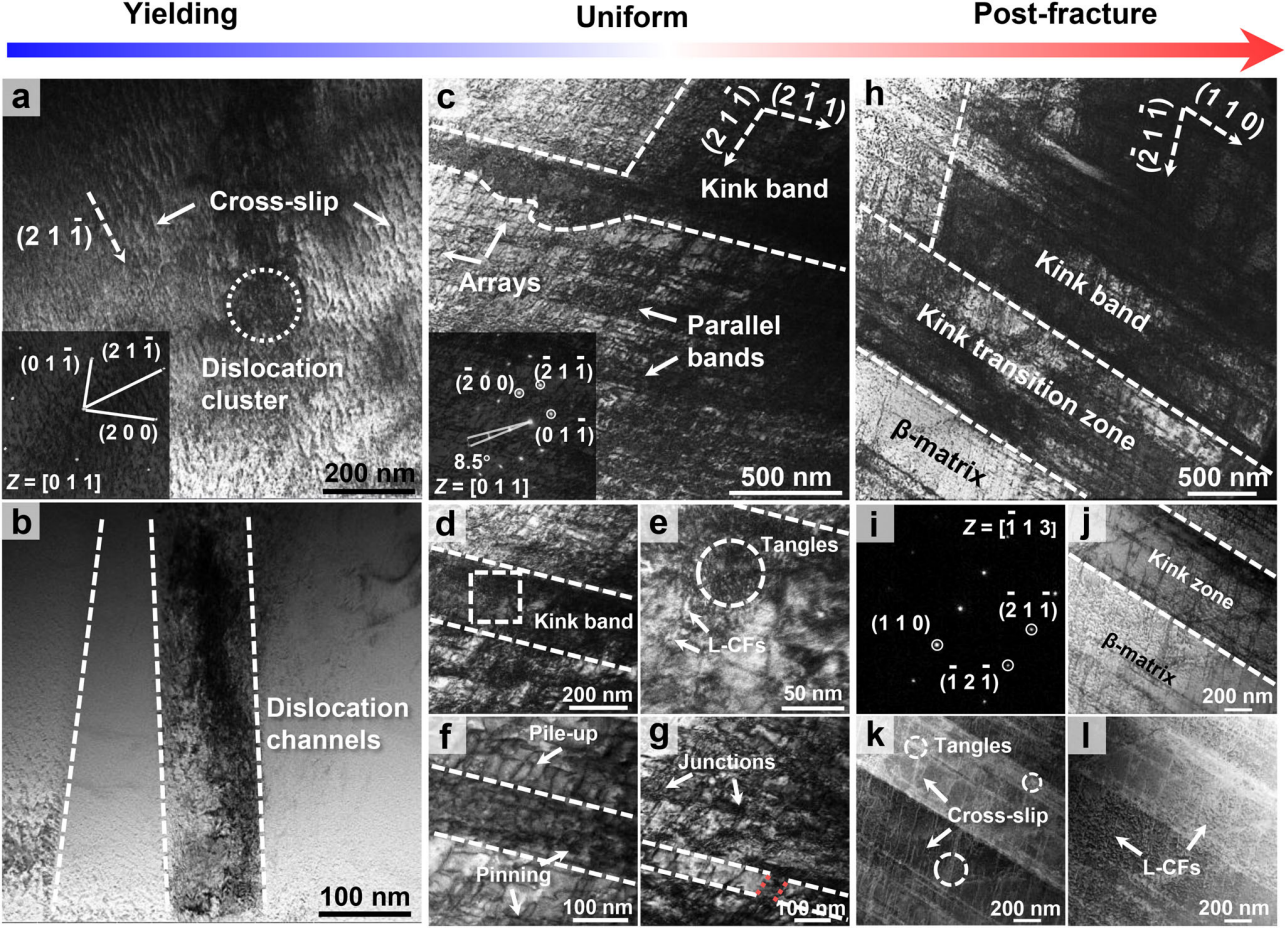
Stage‐dependent TEM/STEM evidence for kink‐band‐mediated deformation in the Hf_10_Ta_5_Sn_5_ alloy. **a, b**, TEM images at the yielding stage, showing cross‐slip, dislocation clustering, and localized dislocation channels. The inset in **a** shows the diffraction pattern from the β matrix. **c–g**, TEM/BF‐STEM images during uniform deformation, showing kink‐band formation, parallel bands and dislocation interactions, including arrays, pile‐up, pinning, tangles and junctions associated with L‐CFs. The inset in **c** shows rotated diffraction spots from the kink‐band region. **h**, TEM image of the post‐fracture region, showing a well‐developed kink‐zone structure. **i**, Diffraction pattern acquired from the kink‐transition zone in **h**, showing no apparent spot rotation. **j–l**, BF‐STEM, DF‐STEM and HAADF‐STEM images of the post‐fracture region, respectively, showing the kink‐zone/β‐matrix region, cross‐slip, dislocation tangles and L‐CFs. Here, the kink zone denotes a kink band together with its adjacent transition zone.

During the uniform‐deformation stage (∼10% engineering strain), deformation heterogeneity becomes much more pronounced. IPF and GROD maps show extensive intragranular color variations, low‐angle banded features, and localized orientation‐gradient zones within individual grains (Figure 4a,b; Figure S8e–h). These bands are confined within grains rather than cutting across multiple grains, indicating crystallographically organized orientation changes rather than non‐crystallographic macroscopic shear localization. Representative banded regions selected from the uniform‐deformation state show different degrees of kink‐band development (Figure 4c,f,i). Nearly parallel low‐angle bands with small point‐to‐point misorientations correspond to incipient kink bands (Figure 4c,d), whereas sharper orientation gradients and larger localized misorientation peaks identify more developed kink bands (Figure 4f,g,i,j). The disorientation‐angle distributions are dominated by low‐ to medium‐angle rotations rather than high‐angle misorientations associated with mechanical twinning. Together with their intragranular confinement and crystallographically organized morphology, these orientation features indicate the formation of kink bands during the uniform‐deformation stage, rather than conventional shear bands or mechanical twins [21, 43].

Pole‐figure analyses further support the kink‐band character of the banded regions formed during uniform deformation. The {110}, {112} and {541} pole figures show pole clustering mainly around <110>‐ or <112>‐type directions (Figure 4e,h,k), consistent with kink‐band‐related lattice rotation involving cooperative a/2<111>‐type dislocation glide in the β‐BCC lattice [57]. TEM/STEM observations provide corresponding defect‐level evidence. Parallel bands, dense dislocation arrays, and pronounced dislocation tangles are observed within locally rotated regions (Figure 5c–e). The diffraction pattern acquired from a kink‐band region shows rotated diffraction spots with an angular deviation of approximately 8.5°, confirming local lattice rotation at the TEM scale (Figure 5c). The consistency between mesoscale EBSD orientation gradients and local TEM diffraction evidence indicates that kink‐band formation during uniform deformation is not an isolated local TEM feature. In the examined uniform‐deformation regions, the band traces are mainly indexed on {211}‐type planes, crystallographically equivalent to the {112} family, suggesting a strong contribution from {112}<111>‐type slip to kink‐band formation at this stage.

The uniform‐deformation microstructures provide experimental evidence for a spatial association between CF‐related chemical/strain heterogeneity and dislocation interactions. Bright‐field STEM (BF‐STEM) images show dislocation pile‐up, pinning, and junction formation near kink‐band/β‐matrix regions (Figure 5f,g), while L‐CFs are observed close to defect‐rich regions (Figure 5e). In addition to locally accumulated dislocations, dislocation channels and cross‐slip traces extend through the surrounding β matrix and across banded regions, suggesting that plastic strain is not confined only within the kink band but can also be transferred to adjacent regions. These observations indicate that the chemical and strain heterogeneity identified in the as‐cast alloy does not simply impose a uniform resistance to dislocation motion [58]. Instead, these observations are consistent with a spatially heterogeneous matrix that favors dislocation pinning, pile‐up, tangling, and junction formation, while allowing dislocation rearrangement and cross‐slip‐assisted strain redistribution [59, 60, 61]. This interpretation is consistent with in situ TEM observations of LCF‐induced dislocation pinning and local double cross‐slip in BCC HfNbTiZr [56], as well as atomistic simulations showing that non‐random local chemical environments roughen the dislocation energy landscape and raise activation barriers for dislocation motion [62]. Such localized dislocation accumulation and strain transfer are consistent with the development of orientation gradients and local lattice rotation associated with kink‐band formation during uniform deformation.

After fracture, the banded deformation substructure becomes more developed. EBSD maps of the post‐fracture region show stronger intragranular orientation gradients, higher GROD contrast, and more pronounced banded features than those observed at yielding and during uniform deformation (Figure S8i–l). Detailed EBSD analysis further reveals a spectrum of kink‐band‐related structures, from low‐angle parallel bands to sharper rotated bands with larger localized misorientation peaks and pole clustering around <110>/<112>‐type directions (Figure S9). These observations indicate that kink‐band‐related lattice rotation continues to develop with increasing strain.

TEM/STEM observations clarify the internal structure of these post‐fracture kinked regions. Here, the kink zone is defined as the combined region consisting of a kink band and its adjacent kink‐transition zone (Figure 5h). The kink band denotes the strongly rotated, dislocation‐rich banded region characterized by clear lattice rotation and intense dislocation accumulation. The kink‐transition zone denotes the intermediate region between the kink band and the surrounding β matrix. In terms of orientation relationship, it remains crystallographically continuous with the β matrix and shows little or no apparent diffraction‐spot rotation compared with the kink‐band interior. In terms of the degree of deformation, it is characterized by less concentrated dislocation accumulation but more frequent cross‐slip traces than the kink band, suggesting a strain‐transfer role between the rotated band and the surrounding matrix.

In the post‐fracture microstructure, the kink band, kink‐transition zone, and β matrix coexist within the same grain (Figure 5h,j). The diffraction pattern acquired from the kink‐transition zone retains β‐BCC reflections without apparent spot rotation (Figure 5i), confirming that this region has not evolved into a fully rotated kink band but instead bridges the rotated kink band and the surrounding matrix. BF‐STEM, dark‐field STEM (DF‐STEM) and HAADF‐STEM images further show dense dislocation tangles, cross‐slip traces and L‐CFs in the kink‐zone/β‐matrix region (Figure 5j–l). Compared with the uniform‐deformation state, the post‐fracture kinked regions show broader participation of both {112}‐type and {110}‐type band traces, suggesting that deformation evolves from predominantly {112}‐associated kink‐band formation during uniform deformation toward a more developed kink‐zone structure involving multiple BCC slip families at higher strain.

Post‐fracture EBSD maps of the wider Ti–Zr–Nb–Hf–Ta(–Sn) alloy series show band‐like deformation features, local lattice distortion, and dense networks of low‐ and medium‐angle boundaries (Figure S10). The 2–10° boundaries account for a major fraction of the measured local boundary population in these mapped regions, indicating that deformation‐induced lattice rotation is largely accommodated through distributed low‐angle substructures rather than only through abrupt high‐angle boundaries. Such distributed misorientation structures are consistent with progressive strain partitioning and local compatibility accommodation, which can help sustain plastic flow by avoiding premature localization into a single catastrophic shear path. These maps are therefore used as qualitative evidence that kink‐band‐related deformation is a recurring plasticity mode in this β‐BCC alloy platform, rather than as a quantitative comparison of kink‐band density among compositions.

The comparison between the Sn‐free Hf_10_Ta_10_ parent alloy and the Sn‐containing Hf_10_Ta_5_Sn_5_ alloy further clarifies the role of Sn. Hf_10_Ta_10_ also shows deformation‐induced defect structures and local kink‐band‐related features, indicating that the parent β‐BCC framework is intrinsically compatible with kink‐band plasticity (Figure S11a–d). However, Hf_10_Ta_5_Sn_5_ exhibits more pronounced L‐CFs in the as‐cast state, clearer dislocation‐channel formation at yielding, and more organized kink‐band‐mediated deformation during uniform deformation and after fracture (Figure S11e–h). Thus, Sn does not create kink‐band plasticity from scratch. Rather, it amplifies local chemical and strain heterogeneity, strengthening the coupling among chemical fluctuations, dislocation interactions, and kink‐band development. This stage‐dependent evolution provides the experimental basis for the mechanism proposed in Figure 6.

**FIGURE 6:**
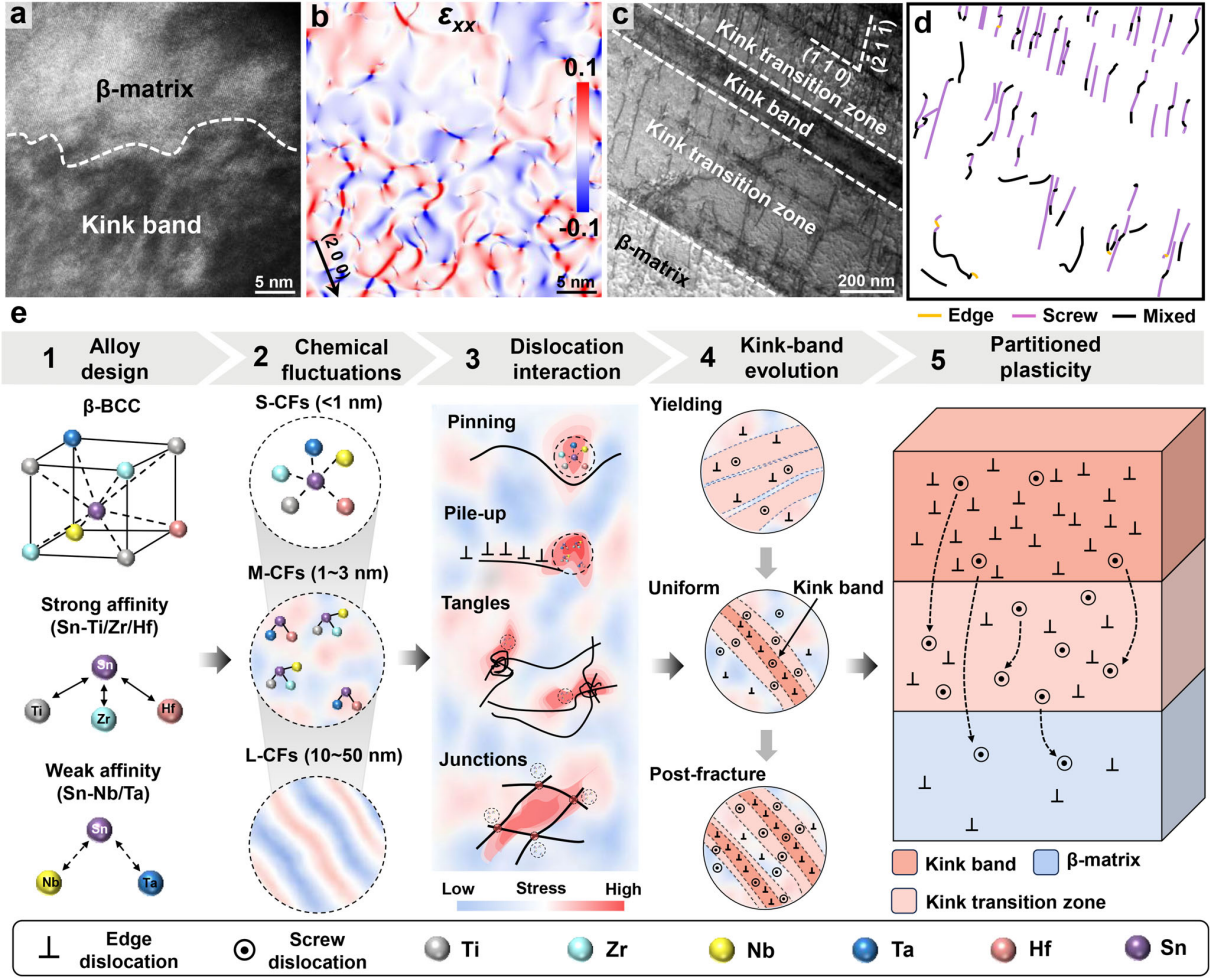
Mechanism of Sn‐induced hierarchical chemical fluctuations and kink‐band‐mediated partitioned plasticity in the Hf_10_Ta_5_Sn_5_ alloy. **a**, HRTEM image showing the interface between a kink band and the adjacent β matrix. **b**, GPA strain map of ε*_xx_* obtained from **a**, showing nanoscale lattice‐strain heterogeneity near the interface. **c**, BF‐STEM image acquired under a two‐beam condition using 𝒈 = (110), showing the post‐fracture banded deformation substructure. **d**, Schematic dislocation‐type map of the corresponding region, showing the coexistence of edge, screw, and mixed dislocations. **e**, Schematic of the proposed CF–dislocation–kink‐band coupling mechanism. Within this framework, the associated chemical–strain heterogeneity can modulate dislocation pinning, pile‐up, tangling, and junction formation. These dislocation interactions facilitate kink‐band‐related lattice rotation and subsequent kink‐band development at higher strain, leading to spatially partitioned plasticity across the banded region and surrounding matrix.

### 2.4 Sn‐Enabled Property Synergy and Cytocompatibility

The above results support a mechanistic link between Sn‐induced hierarchical chemical fluctuations, dislocation interactions, and kink‐band‐mediated plasticity. At the kink‐band/β‐matrix interface, high‐resolution TEM imaging reveals a structurally continuous β‐BCC lattice without a sharp phase boundary (Figure 6a). However, the kink band is not simply a contrast feature. The corresponding GPA strain map shows alternating tensile and compressive strain fields near the interface (Figure 6b), indicating pronounced nanoscale lattice‐strain heterogeneity and local strain concentration. Such alternating strain contrast is consistent with strain fields generated by accumulated dislocations, particularly edge dislocations containing extra half‐planes, which can produce adjacent tensile and compressive lattice distortions [31]. Together with the dense dislocation structures observed in kink‐band regions, these strain fields indicate that kink bands act as defect‐rich strain‐accommodation regions, where plastic deformation is stored through dislocation accumulation and lattice distortion. Thus, kink‐band‐mediated deformation develops within the retained β‐BCC matrix, rather than through phase‐boundary‐mediated deformation.

Two‐beam TEM analysis further clarifies the dislocation character in the post‐fracture banded region. The two‐beam image acquired with a diffraction vector of 𝒈 = (110) shows dense dislocation structures distributed across the kink band, kink‐transition zone, and adjacent β matrix (Figure 6c). The extracted dislocation‐type map reveals a complex population of edge, screw, and mixed dislocations (Figure 6d). Together with the 𝒈·𝒃 invisibility analysis in Figure S12, these observations indicate that deformation is mainly mediated by a/2<111>‐type dislocations in the β‐BCC lattice. Combined with the strain‐field and stage‐resolved TEM observations above, this dislocation population supports a scenario in which edge‐character dislocation accumulation promotes local lattice rotation, while screw and mixed dislocations enable cross‐slip‐assisted rearrangement and strain transfer across the kink band, kink‐transition zone, and surrounding β matrix. The coexistence of different dislocation characters therefore provides a defect‐level basis for both localized lattice rotation and plastic‐strain redistribution. Further local TEM/HRTEM and GPA analyses of the post‐fracture state reveal a locally pinned dislocation associated with nanoscale fluctuation contrast and pronounced strain heterogeneity (Figure S13). This spatial correlation provides microscopic evidence consistent with CF‐associated nanoscale strain heterogeneity locally impeding and redirecting dislocation motion.

The resulting plasticity is spatially partitioned among three coupled regions. Kink bands act as dislocation‐rich zones that accommodate intense local plastic strain through crystallographic rotation, dislocation storage, and strain concentration. Kink‐transition zones bridge the rotated kink bands and the surrounding β matrix, providing geometrical compatibility and stress redistribution through cross‐slip and localized dislocation rearrangement. The β matrix maintains structural continuity and participates in strain accommodation through more distributed slip activity. Surface slip‐trace analysis further supports the involvement of multiple <111>‐type slip systems: {110}<111>, {112}<111>, and {123}<111> slip traces are progressively activated with increasing strain, while wavy slip becomes more pronounced near fracture (Figure S14). Detailed discussions of CF‐assisted dislocation interactions and kink‐band‐mediated partitioning are provided in Notes S3 and S4.

On this basis, we propose a Sn‐mediated chemical–defect–plasticity coupling mechanism (Figure 6e). Sn introduces pronounced pairwise‐interaction contrast within the Hf_10_Ta_5_Sn_5_ β‐BCC matrix, promoting hierarchical chemical fluctuations across multiple length scales. At the atomic scale, S‐CFs are expected to provide local chemical and strain perturbations that can favor dislocation pinning and heterogeneous dislocation storage. At the nanometer scale, M‐CF‐associated chemical/strain heterogeneity can generate spatial variations in local resistance to glide, favoring dislocation pile‐up, tangling, and junction formation. At larger length scales, L‐CF‐rich regions can provide a mesoscopic chemical–strain landscape that biases dislocation accumulation and local orientation gradients. These scale‐dependent roles represent a mechanistic interpretation of the combined multiscale and stage‐resolved observations rather than independently isolated contributions of each CF scale. Taken together, the stage‐resolved observations support a scenario in which fluctuation‐assisted dislocation interactions facilitate the evolution from localized dislocation activity at yielding toward kink‐band formation during uniform deformation and subsequent kink‐band/kink‐transition‐zone/β‐matrix partitioning at higher strain.

The strength–ductility–modulus synergy in Hf_10_Ta_5_Sn_5_ therefore arises from coupled but distinct contributions. The high yield strength reflects the combined contributions of Sn‐induced atomic‐size/modulus mismatch, grain refinement, and CF‐assisted dislocation interactions. The smaller grain sizes of the Sn‐containing alloys may provide an additional strengthening contribution; however, the substantial strength variation among alloys with comparable grain sizes indicates that grain refinement alone cannot account for the observed composition dependence. The large tensile ductility is sustained by kink‐band‐mediated plasticity, in which kink bands accommodate local strain, kink‐transition zones redistribute stress through dislocation rearrangement and cross‐slip, and the surrounding β matrix preserves structural continuity and plastic compatibility. The low Young's modulus is primarily enabled by metastable‐β electronic‐structure design, which retains a compliant β‐BCC framework, and is further assisted by the low intrinsic modulus and bonding perturbation introduced by Sn. The CF‐associated lattice‐strain heterogeneity may further modulate the effective elastic response through spatially heterogeneous bonding and elastic strain accommodation within the β matrix. The observed property balance is therefore not governed by a single microstructural factor, but by the integration of solid‐solution/chemical‐fluctuation strengthening, kink‐band‐mediated strain partitioning, and metastable‐β low‐modulus design.

In addition to its mechanical performance, Hf_10_Ta_5_Sn_5_ exhibits good cytocompatibility. After 72 h of direct culture, MC3T3‐E1 cells on Hf_10_Ta_5_Sn_5_ show a relative cell viability of 108.0 ± 2.4%, higher than those on commercial Ti–6Al–4V (TC4, 100.1 ± 4.1%) and the Sn‐free Hf_10_Ta_10_ alloy (95.1 ± 3.5%) in the same assay (Figure S15a). Fluorescence staining further reveals dense viable cell coverage on the Hf_10_Ta_5_Sn_5_ surface, indicating favorable cell attachment and survival (Figure S15b–e). These results demonstrate the good cytocompatibility of Hf_10_Ta_5_Sn_5_ and support its potential as a low‐modulus load‐bearing biomedical alloy.

In summary, this work advances Sn‐induced hierarchical chemical fluctuations as a compositional route for guiding kink‐band‐mediated plastic deformation in β‐BCC MPEAs. The combined observations support a chemical–defect–plasticity coupling mechanism: contrasting Sn–element interactions generate hierarchical chemical fluctuations and associated local strain fields; these heterogeneous fields favor dislocation pinning, pile‐up, tangling, and junction formation; and the resulting defect accumulation facilitates kink‐band formation during uniform deformation and kink‐band‐mediated partitioning at higher strains. Integrated with metastable‐β low‐modulus design, this mechanism accounts for the unusual combination of high yield strength (∼1.14 GPa), large tensile elongation (20.3%), and low Young's modulus (52.8 GPa) achieved in the as‐cast Hf_10_Ta_5_Sn_5_ alloy. The favorable cytocompatibility results further support the potential applications of this alloy as load‐bearing biomedical materials. More broadly, these findings demonstrate that chemical‐heterogeneity engineering can guide the stage‐dependent evolution of defect activity toward kink‐band‐mediated partitioned plasticity in BCC MPEAs, providing a design principle for achieving strength–ductility–modulus synergy.

## 3 Methods

### 3.1 Alloy Preparation

The Sn‐containing alloys, Ti_35_Zr_35_Nb_10_Hf_7.5_Ta_10_Sn_2.5_ (Hf_7.5_Ta_10_Sn_2.5_), Ti_35_Zr_35_Nb_10_Hf_5_Ta_10_Sn_5_ (Hf_5_Ta_10_Sn_5_), Ti_35_Zr_35_Nb_10_Hf_10_Ta_7.5_ Sn_2.5_ (Hf_10_Ta_7.5_Sn_2.5_), Ti_35_Zr_35_Nb_10_Hf_10_Ta_5_Sn_5_ (Hf_10_Ta_5_Sn_5_) and Ti_35_Zr_35_Nb_10_Hf_7.5_Ta_7.5_Sn_5_ (Hf_7.5_Ta_7.5_Sn_5_) (at. %), were prepared by arc melting Ti, Zr, Nb, Hf, Ta and Sn metals with purities higher than 99.95 wt.% in a water‐cooled copper crucible under a high‐purity argon atmosphere. Before melting, a Ti getter was repeatedly melted to reduce residual oxygen in the chamber. Each ingot was re‐melted at least six times with intermediate flipping to improve chemical homogeneity, and was then gravity‐cast into a water‐cooled copper mold to obtain rectangular ingots with nominal dimensions of 10 × 10 × 60 mm^3^.

### 3.2 Mechanical Testing

Flat dog‐bone‐shaped tensile specimens were sectioned from the as‐cast ingots by electrical discharge machining. The nominal gauge dimensions were 15 mm in length, 3 mm in width, and 1 mm in thickness. Room‐temperature uniaxial tensile tests were performed using a ZwickRoell Z20 universal testing machine at a nominal strain rate of 5 × 10^−4^ s^−1^ in accordance with ASTM E8/E8M‐24. Engineering strain was measured using a clip‐on extensometer with a gauge length of 10 mm, and Young's modulus was determined from the initial linear elastic region following ASTM E111‐17. At least three specimens were tested for each alloy composition, and the reported values are presented as mean ± s.d. For stage‐resolved microstructural characterization, additional interrupted tensile tests were performed on the Hf_10_Ta_5_Sn_5_ alloy to obtain specimens at the yielding stage (∼2% engineering strain) and the uniform‐deformation stage (∼10% engineering strain), besides the post‐fracture specimen. True stress and true strain were converted from the engineering stress–strain data, and the strain‐hardening rate was calculated as Θ = *d*σ_true_/*d*ε_true_.

### 3.3 Microstructural Characterization

The phase constitution, microstructure, elemental distribution, and grain morphology were examined using a field‐emission scanning electron microscope (FE‐SEM, TESCAN GAIA3) equipped with an Oxford electron backscatter diffraction (EBSD) detector and an energy‐dispersive X‐ray spectroscopy (EDS) system. EBSD data were analyzed using AZtecCrystal 3.1 (Oxford Instruments) and ATEX v5.12 software. For SEM/EBSD observation, rectangular specimens (10 × 5 × 1.5 mm^3^) were mechanically ground using SiC abrasive papers, followed by electropolishing in a 10 vol.% HClO_4_ + 90 vol.% C_2_H_5_OH electrolyte. The submicron‐scale microstructure was characterized using a transmission electron microscope (TEM, Talos F200X G2, Thermo Fisher Scientific) operated at 200 kV. Atomic‐resolution high‐angle annular dark‐field scanning transmission electron microscopy (HAADF‐STEM) was performed on a spherical aberration–corrected microscope (Spectra 300, Thermo Fisher Scientific) operated at 300 kV to analyze lattice defects and local CFs. TEM foils of 3 mm in diameter were sectioned from the as‐cast specimens by electrical discharge machining, mechanically thinned to ∼30 µm, and subsequently ion milled using a precision ion polishing system (PIPS II, Gatan 695).

### 3.4 Atom Probe Tomography

Atom probe tomography (APT) was performed on the as‐cast Hf_10_Ta_5_Sn_5_ alloy using a CAMECA INVIZO 6000 instrument for 3D near‐atomic‐scale chemical quantification. APT specimens were prepared by Ga^+^ focused ion beam (FIB) milling in a FEI Versa 3D DualBeam system, followed by a final cleaning step at 5 kV and 48 pA to minimize ion‐induced damage. The analyses were conducted in laser‐pulsed mode using a deep‐ultraviolet laser (257.7 nm) at a pulse repetition rate of 200 kHz. A pulse energy of 12.5–20 pJ was used to maintain a Nb^3+^/Nb^2+^ charge‐state ratio of approximately 2.0. The specimens were held at 40 K under an ultra‐high vacuum of ∼5 × 10^−11^ Torr. The datasets were reconstructed and analyzed using the Image Visualization and Analysis Software (IVAS 6.3).

### 3.5 First‐Principles Calculations

To complement the experimental observations, density functional theory (DFT) calculations were performed using the Cambridge Serial Total Energy Package (CASTEP), based on the plane‐wave pseudopotential approach [63]. The electronic exchange‐correlation interactions were described by the Perdew‐Burke‐Ernzerhof (PBE) functional within the generalized gradient approximation (GGA) [64]. Special quasi‐random structure (SQS) models representing the MPEAs were generated using VASPKIT v1.5.1 [65, 66, 67]. Each SQS supercell contained 40 atoms, ensuring statistical randomness and compositional fidelity. A plane‐wave energy cutoff of 770 eV and a 2 × 2 × 2 Monkhorst‐Pack k‐point mesh were employed for Brillouin zone sampling. The electronic self‐consistent field (SCF) convergence criterion was set to 5.0 × 10^−6^ eV per atom. Full structural relaxation was performed until the residual atomic forces were below 0.01 eV·Å^−1^.

### 3.6 Cytocompatibility Evaluation

The in vitro cytocompatibility of the Hf_10_Ta_5_Sn_5_ alloy was evaluated using MC3T3‐E1 mouse pre‐osteoblast cells, taking commercial Ti–6Al–4V (TC4) and Hf_10_Ta_10_ alloys as comparison groups. The cells were cultured in high‐glucose Dulbecco's modified Eagle's medium supplemented with 10% fetal bovine serum and 1% penicillin–streptomycin at 37°C in a humidified atmosphere containing 5% CO_2_. Specimens with dimensions of 10 × 10 × 1 mm^3^ were ground, polished, ultrasonically cleaned, and sterilized by autoclaving at 121°C for 20 min. All specimens were placed with the polished surface facing upward for direct cell seeding.

Cell viability was measured using a Cell Counting Kit‐8 (CCK‐8) assay. MC3T3‐E1 cells in the logarithmic growth phase were seeded onto the specimen surfaces in 48‐well plates at a density of 1.5 × 10^4^ cells per well and cultured for 72 h. Cells cultured without alloy specimens were used as the control group. After culture, the medium was removed, and the wells were washed three times with phosphate‐buffered saline. Then, 500 µl of culture medium containing 10% CCK‐8 reagent was added to each well and incubated for 2 h. Subsequently, 400 µl of the solution from each well was transferred to a new plate, and the absorbance was measured at 450 nm using a microplate reader. Relative cell viability was calculated as [68]

(1) $$\begin{array}{c} Relative\;cell\;viability\;\left(\%\right)=\frac{OD_{\mathrm{sample}}-OD_{\mathrm{blank}}}{OD_{\mathrm{control}}-OD_{\mathrm{blank}}}\times 100\% \end{array}$$

Here, *OD*_sample_, *OD*_control_ and *OD*_blank_ represent the optical density values of the specimen group, control group, and blank group, respectively.

Live/dead fluorescence staining was used to observe cell survival and distribution on the specimen surfaces. MC3T3‐E1 cells were seeded onto the specimens at a density of 6 × 10^4^ cells per dish and cultured for 72 h. After rinsing with phosphate‐buffered saline, the cells were stained with Calcein‐AM/propidium iodide solution for 15 min at room temperature in the dark, rinsed three times, and imaged using a fluorescence microscope.

Data are presented as mean ± s.d. from three replicates. Statistical significance was evaluated using one‐way analysis of variance followed by pairwise Student's t‐tests, with P < 0.05 considered statistically significant. “n.s.” denotes no significant difference. The corresponding cell‐viability and fluorescence‐staining results are shown in Figure S15.

## Author Contributions

**Jiakun Wang**: methodology, software, data curation, investigation, formal analysis, visualization, writing – original draft. **Jiayao Zhong**: methodology, investigation. **Jinxi Guo**: investigation, methodology. **Jonathan Aristya**: methodology, investigation. **Minghang Zhou**: methodology, investigation. **Liang Zhu**: investigation, methodology. **Xipeng Tan**: formal analysis, resources, writing – review and editing. **Jinfu Li**: conceptualization, supervision, validation, funding acquisition, project administration, resources, writing – review and editing.

## Conflicts of Interest

The authors declare no Conflicts of Interest.

## Supporting information




**Supporting File**: advs78661‐sup‐0001‐SuppMat.pdf.

## Data Availability

The data that support the findings of this study are available from the corresponding author upon reasonable request.
