# Supplementary material for: Hierarchical Chemical Fluctuations Promote Exceptional Strength–Ductility–Modulus Synergy in Biomedical BCC Multi‐Principal‐Element Alloys
Source: Adv Sci (Weinh). 2026 Nov 9:e78661. Online ahead of print. doi: 10.1002/advs.78661 (PMC13748776; doi:10.1002/advs.78661)
Supplement: Supplementary file 1 [file ADVS-9999-e78661-s001.pdf]

Supplementary Information

Hierarchical chemical fluctuations promote exceptional strength–ductility–modulus synergy in biomedical BCC multi-principal-element alloys

Jiakun Wang [1], Jiayao Zhong [1], Jinxi Guo [2], Jonathan Aristya [2], Minghang Zhou [1], Liang Zhu [1], Xipeng Tan [2*], Jinfu Li [1*]

[1] State Key Laboratory of Metal Matrix Composites, School of Materials Science and Engineering, Shanghai Jiao Tong University, Shanghai, 200240, China

[2] Department of Mechanical Engineering, National University of Singapore, Singapore, 117575, Singapore

[*] Corresponding author

Email: J.F. Li (jfli@sjtu.edu.cn); X.P. Tan (xptan@nus.edu.sg)

Contents

Supplementary Note 1 | Alloy design strategy 3
Supplementary Note 2 | Origin of Sn-induced hierarchical chemical fluctuations 6
Supplementary Note 3 | Chemical-fluctuation-assisted dislocation evolution and kink-band formation........ 9
Supplementary Note 4 | Kink-band-mediated partitioned plasticity 12

Supplementary Figure 1 | d-electron-guided alloy design 15
Supplementary Figure 2 | Initial microstructures of the as-cast alloys 16
Supplementary Figure 3 | Tensile fracture morphologies 17
Supplementary Figure 4 | HAADF-STEM imaging and STEM-EDS maps 18
Supplementary Figure 5 | Strain analysis of the $Hf_{10}Ta_5Sn_5$ alloy 19
Supplementary Figure 6 | Atomic-scale chemical fluctuations 20
Supplementary Figure 7 | Charge-density difference maps........ 21
Supplementary Figure 8 | Microstructural evolution during tensile deformation 22
Supplementary Figure 9 | EBSD analysis of kink bands........ 23
Supplementary Figure 10 | Post-fracture microstructures 24
Supplementary Figure 11 | Comparative microstructural evolution during deformation........ 25
Supplementary Figure 12 | Two-beam TEM analysis of dislocations........ 26
Supplementary Figure 13 | Nanoscale fluctuation–dislocation interaction 27
Supplementary Figure 14 | Stage-dependent slip-trace evolution 28
Supplementary Figure 15 | Cytocompatibility evaluation........ 29

Supplementary Table 1 | Parameters for alloy-design calculations........ 30
Supplementary Table 2 | Calculated parameters and measured tensile properties........ 31
Supplementary Table 3 | STEM-EDS compositions in the $Hf_{10}Ta_5Sn_5$ alloy 32
Supplementary References........ 33

Supplementary Note 1 | Alloy design strategy

The present alloy series was developed from the Ti–Zr–Nb–Hf–Ta β-BCC multi-principal-element alloy (MPEA) platform. $Ti_{35}Zr_{35}Nb_{10}Hf_{10}Ta_{10}$, hereafter denoted as $Hf_{10}Ta_{10}$, was selected as the parent composition because it retains a single β-BCC matrix while providing a promising as-cast baseline with low Young's modulus, high yield strength and tensile ductility[1, 2]. This parent alloy therefore offers a suitable framework for introducing targeted compositional perturbations while maintaining the β-BCC structure. The design target was twofold: to reduce Young's modulus by tuning the average electronic descriptors and β-phase-stability window, and to engineer local chemical heterogeneity by tailoring pairwise thermodynamic interactions. This strategy aimed to achieve strength–ductility–modulus synergy while exploring whether enhanced chemical heterogeneity could facilitate kink-band formation during uniform deformation, without additional thermomechanical processing.

Sn was selected as the compositional perturbation element because it provides several design-relevant effects within the Ti–Zr–Nb–Hf–Ta matrix. Compared with the transition-metal constituents, Sn has a distinct valence-electron configuration and is therefore expected to modify the bonding character of the β-BCC matrix. More importantly, Sn exhibits strongly element-dependent mixing enthalpies with Ti, Zr, Nb, Hf and Ta. In the Ti–Zr–Nb–Hf–Ta system, most elemental pairs have near-zero or slightly positive mixing enthalpies, whereas Sn has markedly negative mixing enthalpies with the group-IV elements Ti, Zr and Hf but much weaker interactions with the group-V elements Nb and Ta[3] (**Figure 3d**). This interaction contrast provides a thermodynamic basis for enhancing local chemical fluctuations within the retained β-BCC solid solution. In addition, the relatively large atomic radius and low intrinsic stiffness of Sn can introduce atomic-size and modulus mismatches, generating local strain fields that interact with dislocations. The known biocompatibility of Sn in Ti-based biomedical alloys also supports its selection for the present low-modulus alloy design with potential biomedical relevance[4]. The elemental parameters and physical properties used in the design are summarized in **Supplementary Table 1**.

The Sn-containing compositions were designed by keeping the Ti/Zr/Nb contents unchanged and introducing Sn through partial substitution of Hf and/or Ta. This strategy preserves the Ti–Zr–Nb-based β-BCC framework while minimizing unnecessary compositional variables, allowing the effect of Sn-induced perturbation to be evaluated more clearly. Hf and Ta were selected as adjustable components because they are major alloying constituents in $Hf_{10}Ta_{10}$ and strongly influence the bonding character, electronic structure, phase-stability tendency and mechanical response of the parent alloy. Moreover, Hf and Ta belong to different elemental groups and interact differently with Sn, making the Hf/Ta/Sn ratio an effective parameter for tuning both the average electronic descriptors and the local thermodynamic-interaction landscape. This substitution strategy therefore links the average phase-stability design with the local interaction contrast required for generating chemical heterogeneity.

Following this Hf/Ta/Sn substitution strategy, the candidate Sn-containing compositions were screened using the d-electron design theory together with *VEC*. The d-electron descriptors were used to assess whether Sn substitution could move the $Hf_{10}Ta_{10}$ parent alloy toward the metastable β extension while retaining β-BCC compatibility, whereas *VEC* provided an additional indicator of BCC-forming tendency. The design was intended to access a β-BCC-compatible composition window with tunable bonding characteristics and phase-stability tendency, rather than to induce macroscopic phase instability or martensitic transformation. Within this window, the Sn-induced mixing-enthalpy contrast provided the thermodynamic basis for tuning local chemical heterogeneity. Two d-electron descriptors were calculated: the average bond order ($\overline{Bo}$), which represents the average bonding strength between constituent atoms, and the average d-orbital energy level ($\overline{Md}$), which reflects the average d-electron energy state of the alloy. These descriptors were calculated according to[5]

$$\overline{Bo} = \sum c_i Bo_i \tag{1}$$

$$\overline{Md} = \sum c_i Md_i \tag{2}$$

where (c_i), (Bo_i) and (Md_i) are the atomic fraction, bond order and d-orbital energy level of

element (i), respectively. The elemental parameters used for the calculations are listed in **Supplementary Table 1**.

Within the $\overline{Bo}$–$\overline{Md}$ design framework, partial substitution of Hf and/or Ta by Sn shifts the $Hf_{10}Ta_{10}$ parent alloy toward lower $\overline{Bo}$ values and closer to the metastable β extension region (**Supplementary Figure 1**). In Ti-based β alloys, compositions approaching this metastable-β-related region are generally associated with reduced Young's modulus, provided that the β-BCC framework is retained[6]. This consideration was used to guide the low-modulus aspect of the design. Accordingly, two Sn levels were selected to sample different perturbation strengths: 2.5 at.% Sn was used to introduce a mild shift in the electronic descriptors and phase-stability tendency, whereas 5 at.% Sn was used to produce a more pronounced compositional perturbation while avoiding excessive phase destabilization or loss of plastic deformability. Based on this design direction, five Sn-containing compositions were constructed: $Ti_{35}Zr_{35}Nb_{10}Hf_{7.5}Ta_{10}Sn_{2.5}$, $Ti_{35}Zr_{35}Nb_{10}Hf_{5}Ta_{10}Sn_{5}$, $Ti_{35}Zr_{35}Nb_{10}Hf_{10}Ta_{7.5}Sn_{2.5}$, $Ti_{35}Zr_{35}Nb_{10}Hf_{10}Ta_{5}Sn_{5}$ and $Ti_{35}Zr_{35}Nb_{10}Hf_{7.5}Ta_{7.5}Sn_{5}$, hereafter denoted as $Hf_{7.5}Ta_{10}Sn_{2.5}$, $Hf_{5}Ta_{10}Sn_{5}$, $Hf_{10}Ta_{7.5}Sn_{2.5}$, $Hf_{10}Ta_{5}Sn_{5}$ and $Hf_{7.5}Ta_{7.5}Sn_{5}$, respectively. For these designed compositions, the calculated VEC values remain in the range of 4.15–4.20, consistent with their BCC-forming tendency (**Supplementary Table 2**). Their configurational mixing entropies remain relatively high, ranging from approximately 12.32 to 12.50 J/(mol·K), whereas their mixing enthalpies shift from the weakly positive value of the $Hf_{10}Ta_{10}$ parent alloy to more negative values after Sn alloying. These calculations suggest that the designed alloys can retain a solid-solution β-BCC matrix while allowing controlled changes in bonding characteristics, phase-stability tendency and local chemical-interaction landscape. The single β-BCC structure observed in the as-cast alloys is consistent with this phase-window design (**Supplementary Figure 2**). Together, these calculations and structural observations support the use of Sn substitution as a compositional perturbation strategy for balancing modulus, strength and ductility in a β-BCC MPEA framework.

Supplementary Note 2 | Origin of Sn-induced hierarchical chemical fluctuations

The multiscale TEM/STEM observations reveal that the as-cast $Hf_{10}Ta_5Sn_5$ alloy contains chemical fluctuations (CFs) across three characteristic length scales: short-range CFs (S-CFs, <1 nm), medium-range CFs (M-CFs, 1–3 nm) and long-range CFs (L-CFs, 10–50 nm) (**Figure 2**). These features do not correspond to crystallographically ordered precipitates or secondary phases, as indicated by the absence of extra diffraction spots in the corresponding SAED/FFT patterns. Instead, they represent hierarchical chemical fluctuations coupled with local lattice-strain heterogeneity within the retained single β-BCC matrix. Here, we rationalize their likely origin from Sn-induced thermodynamic-interaction contrast, local electronic perturbation and strain accommodation within the chemically disordered β matrix.

A key feature of the present alloy design is the pronounced pairwise mixing-enthalpy contrast introduced by Sn. Sn exhibits strongly negative mixing enthalpies with the group-IV elements Ti, Zr and Hf, with values of −21, −43 and −35 kJ/mol, respectively, whereas its interactions with the group-V elements Nb and Ta are much weaker, with values close to neutral or only weakly negative, −1 and −3 kJ/mol, respectively (**Figure 3d**). Most intra-group pairs, such as Ti–Zr–Hf or Nb–Ta, show values close to zero, whereas several inter-group pairs exhibit slightly positive values. Therefore, Sn does not simply increase the overall chemical affinity among all constituent elements. Rather, it introduces an asymmetric thermodynamic landscape in which Sn preferentially stabilizes Ti/Zr/Hf-rich local coordination environments over Nb/Ta-rich ones. This interaction contrast provides a thermodynamic basis for non-random local chemical redistribution within the β-BCC solid solution.

The charge-density-difference calculations provide an electronic-level explanation for this chemically heterogeneous tendency (**Figure 3e,f and Supplementary Figure 7**). The charge-density maps reveal local charge redistribution around Sn-containing configurations, with charge depletion around Sn and charge accumulation along neighboring transition-metal atoms. These features indicate polarized heteroatomic bonding and local bonding asymmetry. The d-electron-rich elements, especially Nb and Ta, retain relatively strong

metallic bonding within the β matrix, whereas Sn perturbs the local electronic environment through charge redistribution with surrounding Ti/Zr/Hf-rich configurations. Together with the relatively large atomic radius and low intrinsic stiffness of Sn, this electronic perturbation couples local chemical heterogeneity with lattice distortion and thereby promotes spatially heterogeneous strain fields.

APT provides complementary evidence for this non-random chemical landscape (**Figure 3a–c**). The 5 at.% Sn iso-concentration surface and the corresponding one-dimensional concentration profiles show spatially heterogeneous Sn-containing regions and coupled fluctuations of Ti, Zr, Nb, Hf, Ta and Sn. These features should not be interpreted as fully separated compositional clusters or precipitates. Rather, they support nanoscale chemical modulation within a retained β-BCC solid solution, consistent with the HAADF-STEM and STEM-EDS observations.

At the shortest length scale, S-CFs originate from Sn-biased local coordination environments. In a fully random β-BCC solid solution, Sn would sample the surrounding elements without a strong preference. However, the strongly negative Sn–Ti/Zr/Hf mixing enthalpies make Ti/Zr/Hf-containing coordination shells energetically more favorable, whereas the much weaker Sn–Nb/Ta interactions do not provide the same stabilization. This local coordination bias produces atomic-scale deviations from a random elemental distribution. In the HAADF-STEM and atomic-resolution STEM-EDS maps, these deviations appear as sub-nanometer composition and intensity fluctuations among neighboring atomic columns (**Figure 2h–l and Supplementary Figure 6**). Because these Sn-biased local environments also contain atoms with different sizes and intrinsic stiffnesses, they generate local lattice-strain perturbations, as reflected by the atomic-scale GPA strain maps and strain profiles (**Figure 2j and Supplementary Figure 5**). Thus, S-CFs are best understood as atomic-scale chemical-coordination fluctuations coupled with local lattice-strain perturbations, rather than as ordered clusters or precipitate embryos.

At the nanometer scale, M-CFs arise when these atomic-scale coordination fluctuations become spatially correlated over several neighboring atomic columns. The HAADF-STEM

images show diffuse bright–dark contrast modulations with characteristic dimensions of approximately 1–3 nm (**Figure 2d–g**). These M-CFs do not have sharp interfaces or fixed stoichiometry. Instead, they represent nanoscale compositionally modulated domains produced by the spatial correlation of Sn-biased local environments under the constraints of the surrounding β matrix. Bright domains are generally associated with relatively heavier-element-enriched environments involving Hf, Ta and Sn, whereas darker domains are relatively enriched in lighter Ti/Zr/Nb-containing environments. This nanoscale chemical contrast is accompanied by local lattice-strain variations, indicating that M-CFs reflect both chemical-interaction contrast and strain accommodation within the single β-BCC matrix.

At the larger scale, L-CFs appear as ridge-like or stripe-like HAADF contrast features with widths of approximately 10 nm and lengths extending to several tens of nanometers (**Figure 2a and Supplementary Figure 4**). STEM-EDS mapping and local composition analysis show that these bright regions are relatively enriched in Hf, Ta and Sn compared with the surrounding matrix (**Supplementary Table 3**). The absence of additional diffraction spots indicates that these features remain structurally coherent with the β-BCC matrix rather than forming a distinct second phase. Their diffuse interfaces and long-wavelength compositional modulation are consistent with a spinodal-like chemical fluctuation characteristic. However, the present evidence does not require assigning them to a fully developed equilibrium spinodal decomposition process. More conservatively, L-CFs can be described as long-wavelength compositionally modulated domains that arise when chemical-interaction contrast and strain accommodation are correlated over larger distances within the retained β-BCC solid solution.

The hierarchical CFs should therefore be viewed as nested chemical fluctuations across different correlation lengths, rather than as a strictly sequential transformation from S-CFs to M-CFs and then to L-CFs. Atomic-scale Sn-biased coordination produces S-CFs; spatial correlation of these local fluctuations gives rise to M-CFs; and longer-wavelength chemical modulation produces L-CFs.

Supplementary Note 3 | Chemical-fluctuation-assisted dislocation evolution and kink-band formation

The hierarchical CFs discussed in **Supplementary Note 2** create a multiscale chemical–strain landscape in the retained β-BCC matrix. Instead of acting as a uniform solid-solution friction, this landscape introduces local variations in glide resistance, dislocation storage and lattice rotation. Here, we rationalize how S-CFs, M-CFs and L-CFs bias dislocation evolution and create favorable conditions for kink-band formation in the $Hf_{10}Ta_5Sn_5$ alloy.

The stage-resolved EBSD and TEM observations establish the experimental basis for this discussion. Plastic deformation starts heterogeneously at yielding, as indicated by localized dislocation channels and small dislocation clusters (**Figure 5a,b**). During uniform deformation, intragranular orientation gradients and kink-band-related domains appear together with dislocation arrays, pile-ups, tangles and junctions (**Figs. 4 and 5c–g**). At higher strain, these structures further develop into a partitioned kink-zone structure consisting of kink bands, kink-transition zones and relatively less-deformed β-matrix regions (**Figure 5h–l; Supplementary Figure 8**). Thus, the deformation sequence is characterized by a transition from localized dislocation accumulation to kink-band-mediated strain accommodation.

HRTEM and GPA analyses provide local evidence for this defect-mediated interpretation. Alternating tensile and compressive strain fields are observed near kink-band/β-matrix regions (**Figure 6a,b**), consistent with lattice distortions generated by accumulated dislocations, particularly edge dislocations with extra half-planes. Together with the pile-ups, tangles and pinned arrays observed during uniform deformation (Figure 5c–g), these strain fields indicate that kink bands emerge from localized dislocation storage and lattice rotation within the chemically heterogeneous β matrix, rather than from a phase-boundary-mediated mechanism.

The Sn-free $Hf_{10}Ta_{10}$ alloy provides a useful reference for separating the intrinsic deformation mode of the β-BCC platform from the effect of Sn-induced chemical

heterogeneity. $Hf_{10}Ta_{10}$ also exhibits deformation-induced defect structures and kink-band-related features, whereas $Hf_{10}Ta_5Sn_5$ shows more pronounced L-CFs, clearer dislocation-channel formation at yielding and more organized kink-band-associated dislocation structures during uniform deformation (**Supplementary Figure 11**). Thus, Sn should not be viewed as creating kink-band plasticity from scratch; rather, it amplifies the chemical–strain landscape and strengthens CF–dislocation coupling.

Two-beam TEM further confirms that deformation is mediated by typical BCC a/2<111>-type dislocations (**Supplementary Figure 12**). The coexistence of edge, screw and mixed dislocations is consistent with a dual requirement for kink-band formation: localized dislocation accumulation to promote lattice rotation, and cross-slip-assisted dislocation rearrangement to transfer strain across kink-band, kink-transition-zone and β-matrix regions (**Figure 6c,d**). Additional local TEM/HRTEM and GPA analyses further reveal a locally pinned dislocation associated with nanoscale fluctuation contrast and pronounced alternating strain heterogeneity (Supplementary Figure 13). This spatial correlation provides microscopic evidence consistent with nanoscale CF-associated strain heterogeneity locally perturbing dislocation glide.

On this basis, the scale-dependent role of hierarchical CFs can be rationalized as follows.

First, S-CFs act as atomic-scale chemical and lattice-strain perturbations to dislocation motion. As discussed in **Supplementary Note 2**, these S-CFs arise from Sn-biased local coordination environments, bonding asymmetry, atomic-size mismatch and local strain fluctuations. Such short-range perturbations can serve as local obstacles for moving dislocations (**Figure 2h–l; Supplementary Figs. 5 and 6**). They are expected to interact particularly strongly with edge dislocations because the strain fields of edge dislocations couple sensitively with local chemical and strain inhomogeneities[7, 8]. Such atomic-scale perturbations can therefore favor dislocation pinning, local stress build-up and heterogeneous dislocation storage at the earliest stage of plastic deformation. However, S-CFs should not be regarded as isolated “nucleation sites” for kink bands. Instead, they

provide elementary atomic-scale barriers that initiate heterogeneous dislocation accumulation.

Second, M-CFs extend these local perturbations into nanoscale compositionally modulated domains. The HAADF-STEM observations suggest that heavier-element-enriched and relatively lighter-element-enriched regions coexist at the 1–3 nm scale (**Figure 2d–g**). Such nanoscale chemical contrast can create local variations in stiffness, strain energy and glide resistance, thereby modulating dislocation pathways. Dislocations may move more readily through locally lower-resistance regions while being deflected, pinned or accumulated near chemically fluctuating and locally strained domains. The local dislocation-pinning configuration associated with nanoscale fluctuation/strain heterogeneity (**Supplementary Figure 13**), together with the dislocation arrays, pile-ups and junctions observed during uniform deformation (**Figure 5c,f,g**), supports this interpretation. In this sense, M-CFs do not directly "create" kink bands, but precondition the β matrix by organizing dislocations into locally stressed configurations that are more susceptible to local lattice rotation.

Third, L-CFs provide a longer-wavelength chemical–strain landscape that can guide the spatial development of kink-band-related deformation. The ridge-like Hf/Ta/Sn-enriched L-CFs remain coherent with the β-BCC matrix and possess diffuse interfaces rather than sharp phase boundaries (**Figure 2a; Supplementary Figure 4**). Under tensile loading, the stiffness and local strain contrast between L-CF-rich regions and the surrounding matrix can enhance strain incompatibility. Dislocations accumulated at or near these compositionally modulated regions may then generate local orientation gradients. Once the local stress and lattice rotation become sufficiently large, these regions can become favorable locations for kink-band-related lattice rotation and subsequent band development. Thus, L-CFs are best viewed as mesoscopic scaffolds that bias the spatial development of kink-band-related deformation, rather than deterministic templates that fully prescribe kink-band geometry.

Supplementary Note 4 | Kink-band-mediated partitioned plasticity

The EBSD and TEM observations indicate that plastic deformation in the $Hf_{10}Ta_5Sn_5$ alloy is not accommodated by a single homogeneous slip mode. Instead, deformation becomes spatially partitioned among kink bands, kink-transition zones and the surrounding β matrix. Here we discuss the crystallographic basis of kink-band formation and the role of these three regions in coordinating plastic strain. This discussion is not intended to establish a universal hardening model for all BCC alloys, but to rationalize how kink-band-mediated plasticity operates in the present chemically heterogeneous β-BCC alloy.

The active slip systems associated with kink-band formation can be inferred from intragranular misorientation-axis (IGMA) analysis[9]. For a local tilt boundary, the rotation or Taylor axis $\boldsymbol{T}$ is related to the Burgers vector $\boldsymbol{b}$ and the slip-plane normal $\boldsymbol{n}$ according to $\boldsymbol{T} = \gamma \boldsymbol{b} \times \boldsymbol{n}$, where γ is a proportionality constant[9]. In BCC crystals, dislocations generally have a Burgers vector of a/2<111>, and the main slip planes belong to the {110}, {112} and {123} families. Slip on these planes gives rise to characteristic Taylor axes close to <112>, <110> and <541>, respectively. Therefore, the distribution of pole centers and misorientation axes provides a useful crystallographic indicator of the slip systems involved in kink-band-related lattice rotation.

For the $Hf_{10}Ta_5Sn_5$ alloy, the kink-band regions formed during uniform deformation exhibit misorientation angles mainly below 20° and pole centers close to <110> or <112>, indicating that {112}<111> and {110}<111> slips are strongly involved in kink-band formation (**Figure 4**). Similar crystallographic features are observed after fracture, where mature kink-band-related domains retain rotation axes around <110>/<112> and show intragranular orientation gradients characteristic of kink-band lattice rotation (**Supplementary Figure 9**). These observations support the view that kink bands in this alloy arise from cooperative <111>-type dislocation glide and local lattice rotation, rather than from mechanical twinning or conventional shear banding.

The post-fracture EBSD overview further shows composition-dependent band-like deformation features across the Ti–Zr–Nb–Hf–Ta(–Sn) alloy series (**Supplementary Figure**

10). These maps are used as representative qualitative evidence, not as a quantitative measure of kink-band density. Nevertheless, the presence of band-like orientation gradients and low-angle boundary networks across different compositions suggests that kink-band-related deformation is a recurring plasticity mode within this β-BCC alloy platform. In the optimized $Hf_{10}Ta_5Sn_5$ alloy, this mode is coupled with the hierarchical CF landscape and pronounced dislocation storage discussed in **Supplementary Note 3**.

On this basis, the overall plastic response of $Hf_{10}Ta_5Sn_5$ can be interpreted as a kink-band-mediated partitioned plasticity mechanism involving three coupled regions.

First, kink-band zones act as localized strain-accommodation and energy-dissipation regions. TEM/STEM observations show that kink-band interiors contain dense dislocation arrays, pile-ups and tangles, and the selected-area diffraction patterns reveal local lattice rotation relative to the surrounding matrix (**Figure 5c–g**). These features indicate that kink bands accommodate substantial local plastic strain through dislocation accumulation and crystallographic rotation. Importantly, such localization does not immediately lead to fracture. Instead, the kink bands appear to confine intense dislocation activity within selected intragranular regions, thereby allowing plastic strain to be absorbed locally while avoiding uncontrolled propagation of damage throughout the entire grain.

Second, kink-transition zones provide geometrical compatibility and stress redistribution between the kink bands and the surrounding β matrix. These transition zones show moderate orientation gradients and a lower dislocation density than the kink-band interiors, but they contain frequent cross-slip traces and mixed dislocation configurations **(Figs. 5h–l and 6c,d**). Two-beam TEM analyses further reveal the coexistence of edge, screw and mixed a/2<111> dislocations, with screw and mixed dislocations being closely associated with cross-slip and strain transfer (**Supplementary Figure 12**). These observations suggest that kink-transition zones function as strain-bridging regions. They allow dislocations to rearrange, cross-slip and redistribute local stress between the highly rotated kink-band interiors and the less-deformed β matrix, thereby maintaining intragranular compatibility.

Third, the surrounding β matrix serves as a structural framework that preserves grain integrity and helps homogenize macroscopic deformation. Compared with the kink-band interiors, matrix regions generally show lower dislocation density and less severe lattice rotation (**Figure 5j–l**). This does not mean that the matrix is inactive. Rather, the matrix accommodates residual incompatibility through limited slip, cross-slip and local dislocation-channel formation when required. In this way, the β matrix helps smooth strain gradients while avoiding the development of a fully localized shear path. The coexistence of highly deformed kink bands, intermediate transition zones and relatively less-deformed matrix regions therefore enables deformation to be partitioned over multiple spatial regions instead of being concentrated into a single catastrophic band.

The surface slip-trace analysis provides additional evidence for the stage-dependent activation of slip systems (**Supplementary Figure 14**). At the early yielding stage, slip traces are mainly consistent with {110}<111> and {112}<111> slip. During uniform deformation, additional {123}<111> slip traces appear, indicating enhanced slip multiplicity and improved strain compatibility. Near fracture, wavy-slip traces become dominant, consistent with increased screw-dislocation cross-slip and local dislocation rearrangement. This slip-trace evolution agrees with the two-beam TEM evidence for mixed dislocation characters and cross-slip in kink-transition/matrix regions, and further supports a transition from relatively planar slip toward multi-slip and wavy-slip-assisted strain redistribution.

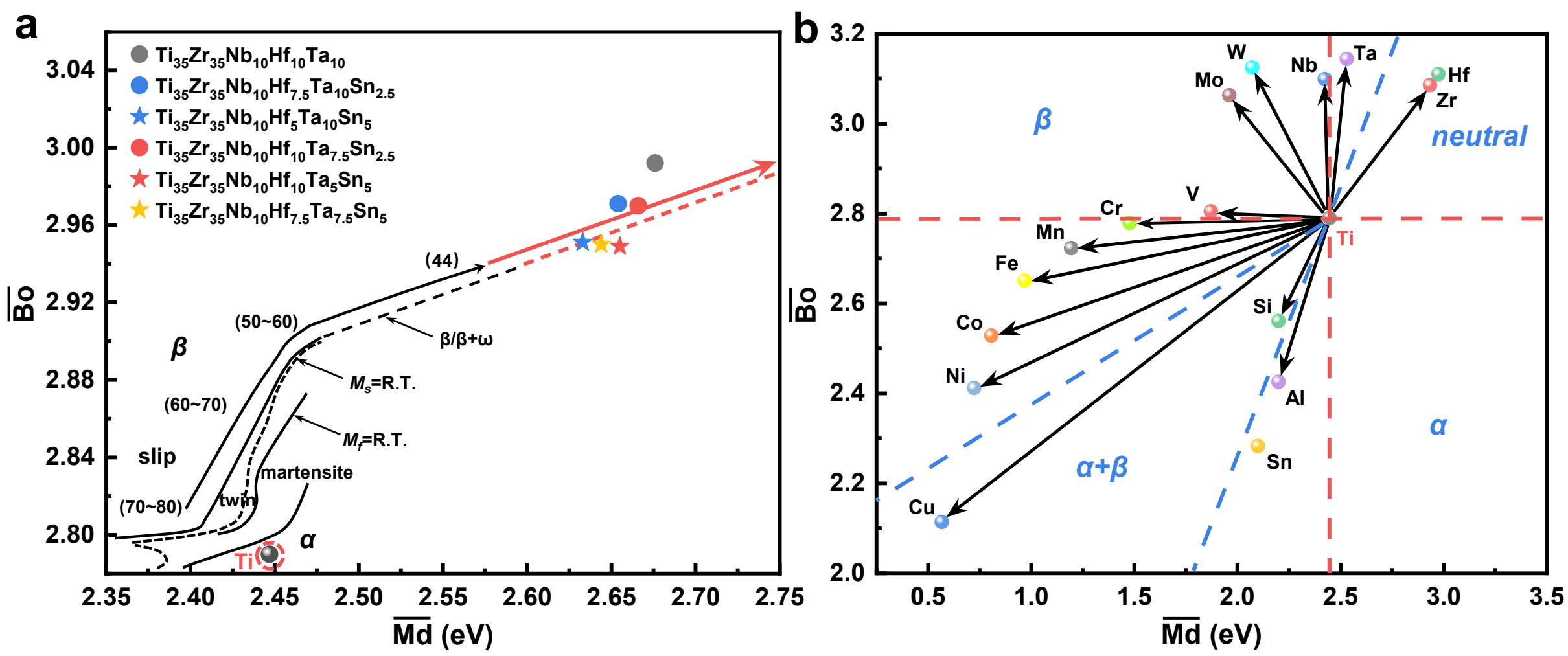

Supplementary Figure 1 | Extension of d-electron-guided metastable-β design to Sn-alloyed Ti–Zr–Nb–Hf–Ta β-BCC MPEAs. **a**, $\overline{Bo}$-$\overline{Md}$ diagram showing the $Hf_{10}Ta_{10}$ parent alloy and designed Sn-containing compositions relative to the conventional Ti-alloy phase-stability and deformation-mode regions. The Ti–Zr–Nb–Hf–Ta(–Sn) MPEAs lie outside the conventional Ti-alloy composition range but follow the metastable-β-related extension of the $\overline{Bo}$-$\overline{Md}$ framework, which is used here to guide low-modulus β-BCC alloy design while retaining β-phase compatibility. **b**, Directional effects of representative alloying elements on $\overline{Bo}$ and $\overline{Md}$ relative to Ti, illustrating how different elements shift the average electronic descriptors and influence α/β phase-stability tendency. Sn shifts the design toward lower $\overline{Bo}$ and $\overline{Md}$, whereas Nb, Ta, Zr and Hf help maintain β-BCC compatibility, providing a compositional tuning framework for Sn-alloyed Ti–Zr–Nb–Hf–Ta β-BCC MPEAs.

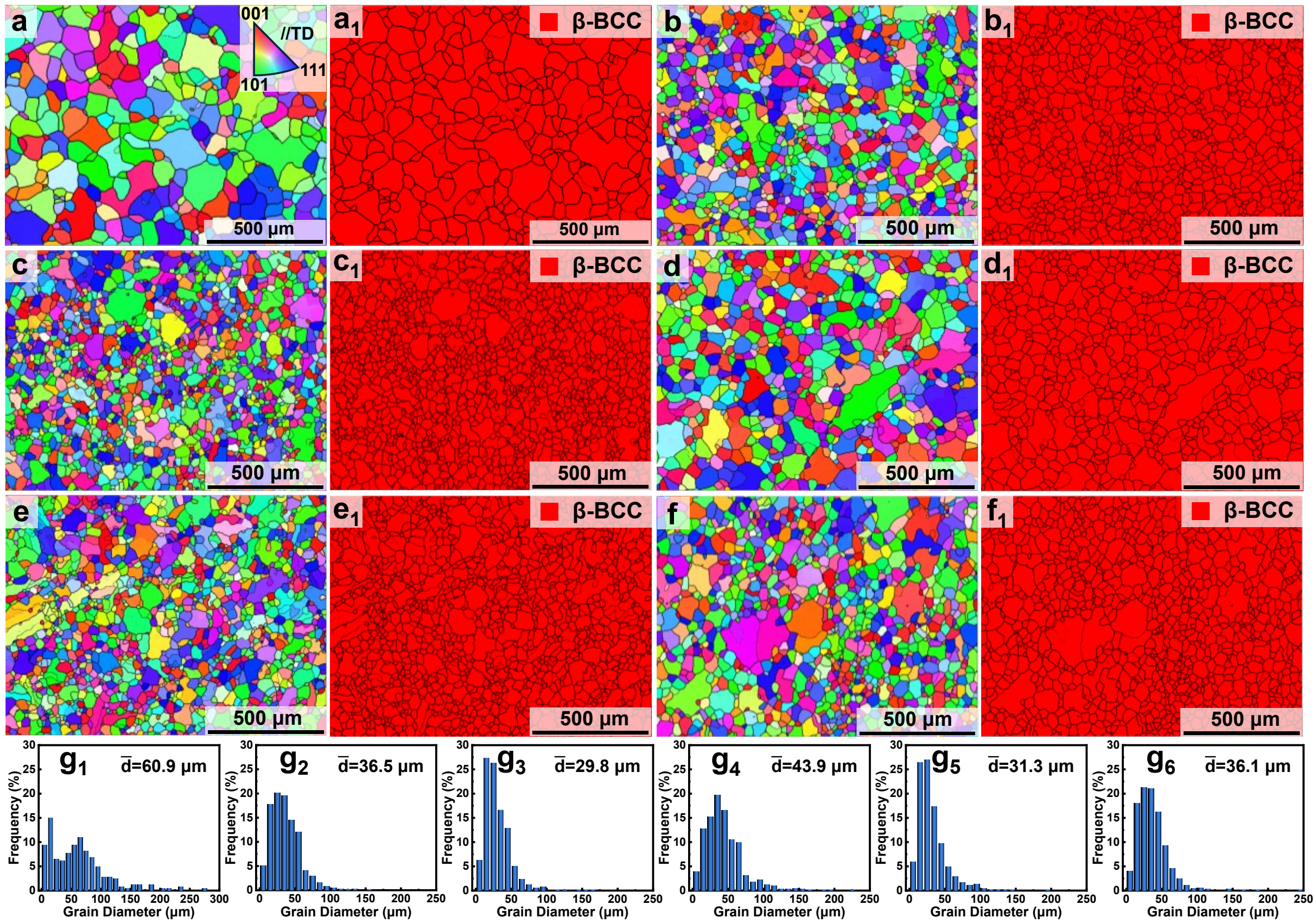

Supplementary Figure 2 | EBSD microstructures and grain-size distributions of the as-cast Ti-Zr-Nb-Hf-Ta(-Sn) alloys. a–f, EBSD inverse pole figure maps of the $Hf_{10}Ta_{10}$, $Hf_{7.5}Ta_{10}Sn_{2.5}$, $Hf_5Ta_{10}Sn_5$, $Hf_{10}Ta_{7.5}Sn_{2.5}$, $Hf_{10}Ta_5Sn_5$ and $Hf_{7.5}Ta_{7.5}Sn_5$ alloys, respectively. **a_1–f_1**, Corresponding EBSD phase maps showing a single β-BCC structure. **g_1–g_6**, Grain-size distributions derived from the corresponding EBSD maps.

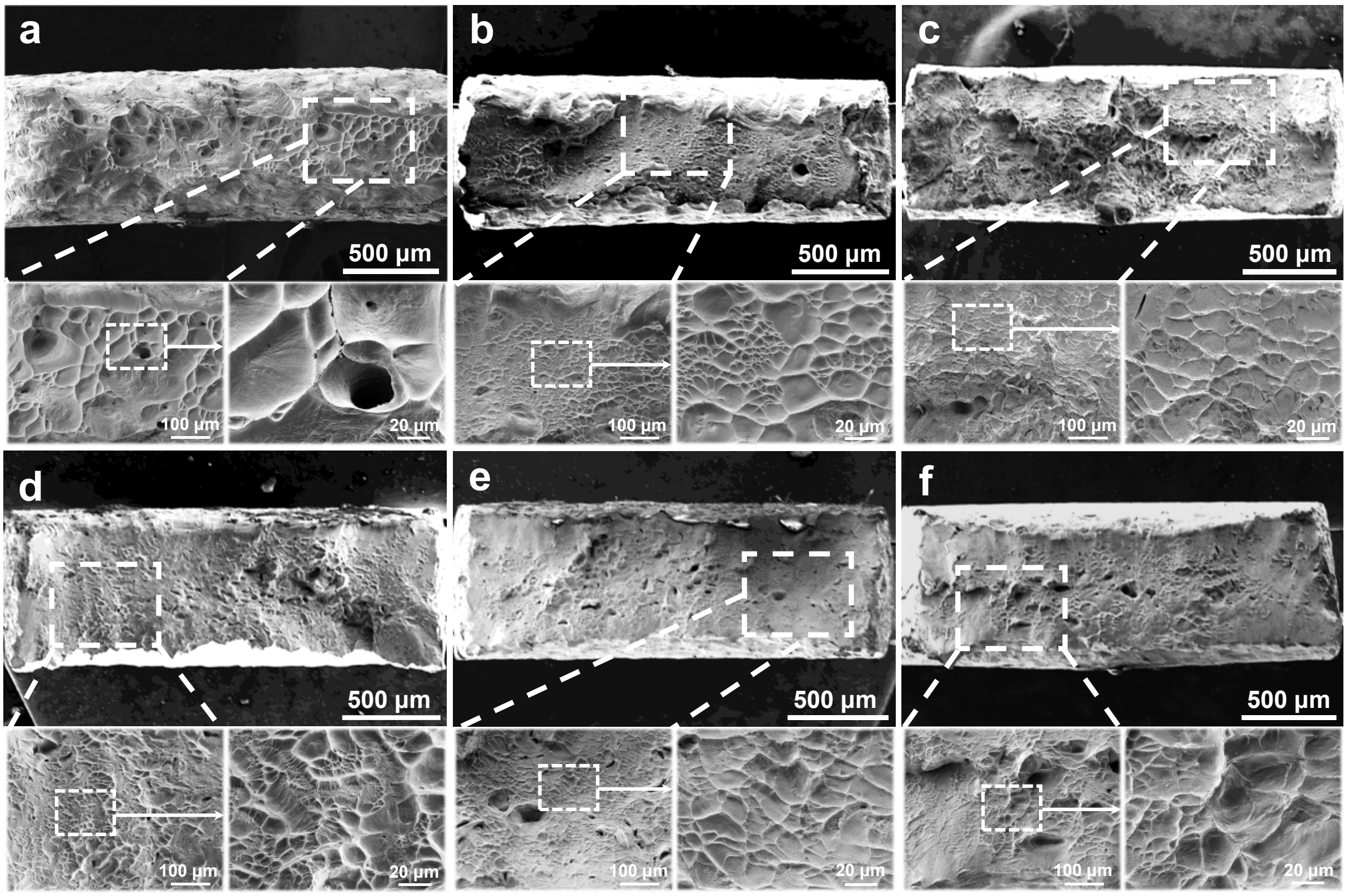

Supplementary Figure 3 | Tensile fracture morphologies of the Ti-Zr-Nb-Hf-Ta(-Sn) alloys. a–f, SEM fracture-surface images of the $Hf_{10}Ta_{10}$, $Hf_{7.5}Ta_{10}Sn_{2.5}$, $Hf_5Ta_{10}Sn_5$, $Hf_{10}Ta_{7.5}Sn_{2.5}$, $Hf_{10}Ta_5Sn_5$ and $Hf_{7.5}Ta_{7.5}Sn_5$ alloys, respectively. The magnified images show the local fracture features within the marked regions.

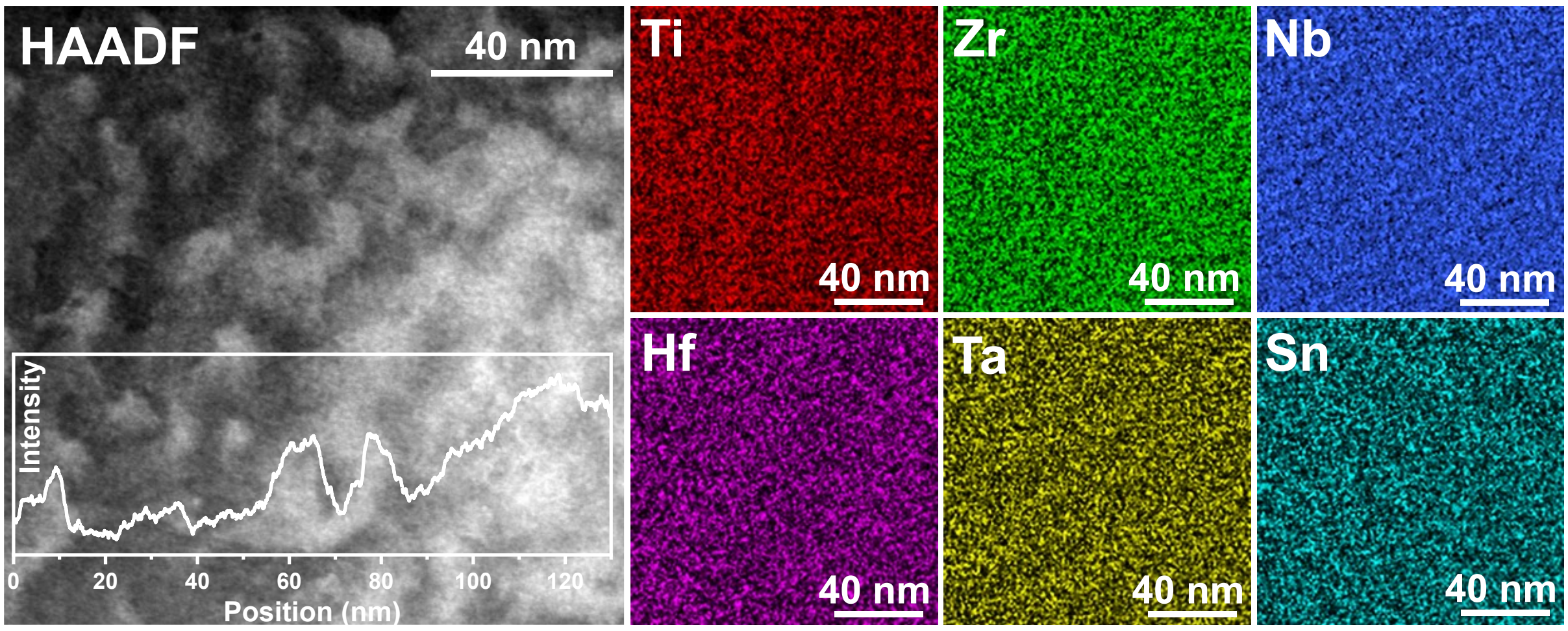

Supplementary Figure 4 | Enlarged HAADF-STEM imaging and STEM-EDS maps of the $Hf_{10}Ta_5Sn_5$ alloy. Enlarged HAADF-STEM image and corresponding STEM-EDS elemental maps of Ti, Zr, Nb, Hf, Ta and Sn acquired from the region shown in Figure 2a. The inset shows the HAADF intensity profile across a region containing L-CFs and the surrounding β matrix, revealing nanoscale Z-contrast fluctuations.

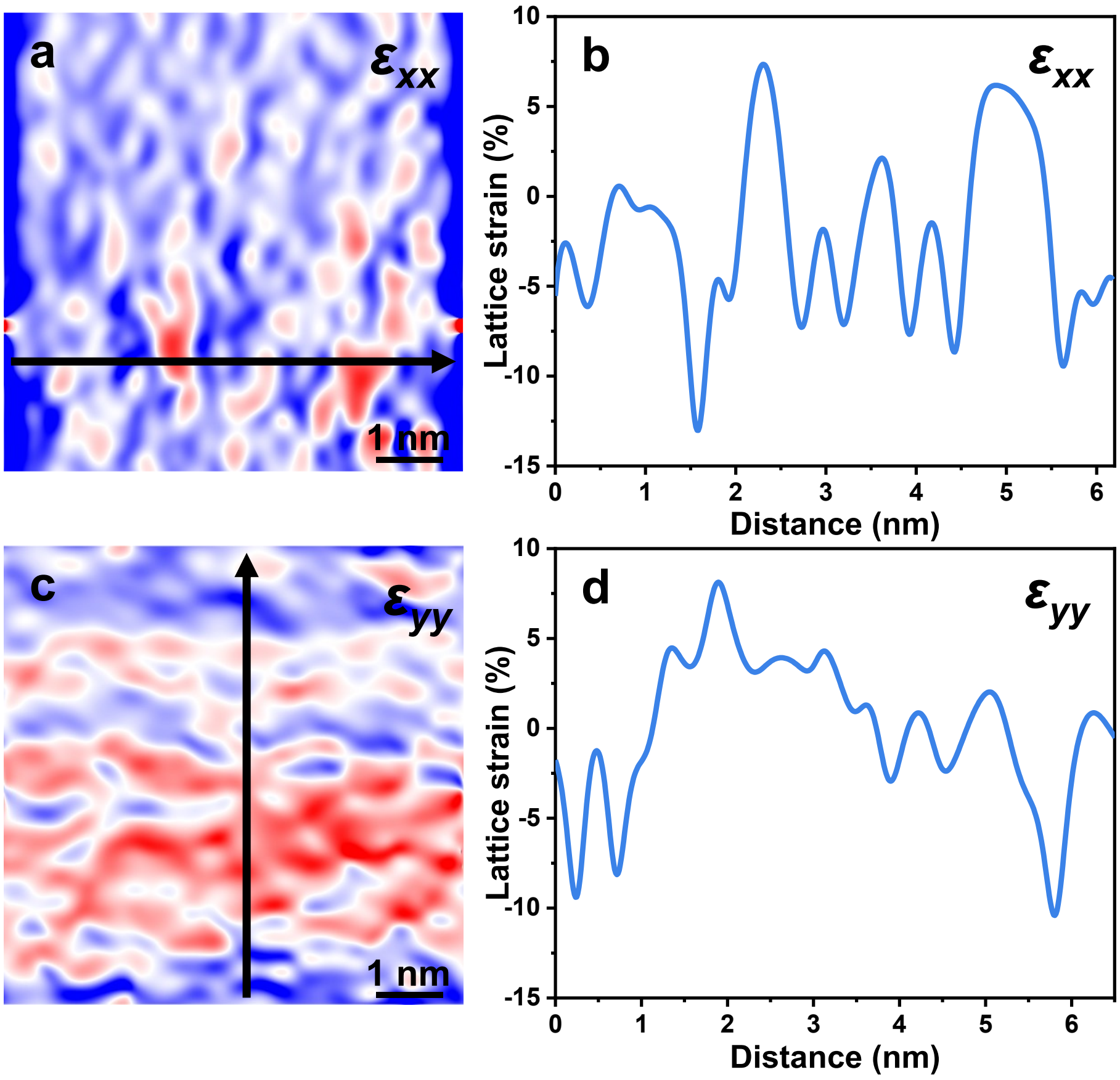

Supplementary Figure 5 | Atomic-scale lattice-strain analysis of the $Hf_{10}Ta_5Sn_5$ alloy. a, c, Geometric phase analysis (GPA) strain maps of ε_{xx} and ε_{yy} acquired from the regions shown in Figure $2j_1$ and Figure $2j_2$, respectively. **b, d**, Corresponding strain profiles measured along the arrows in **a** and **c**, showing nanoscale lattice-strain fluctuations.

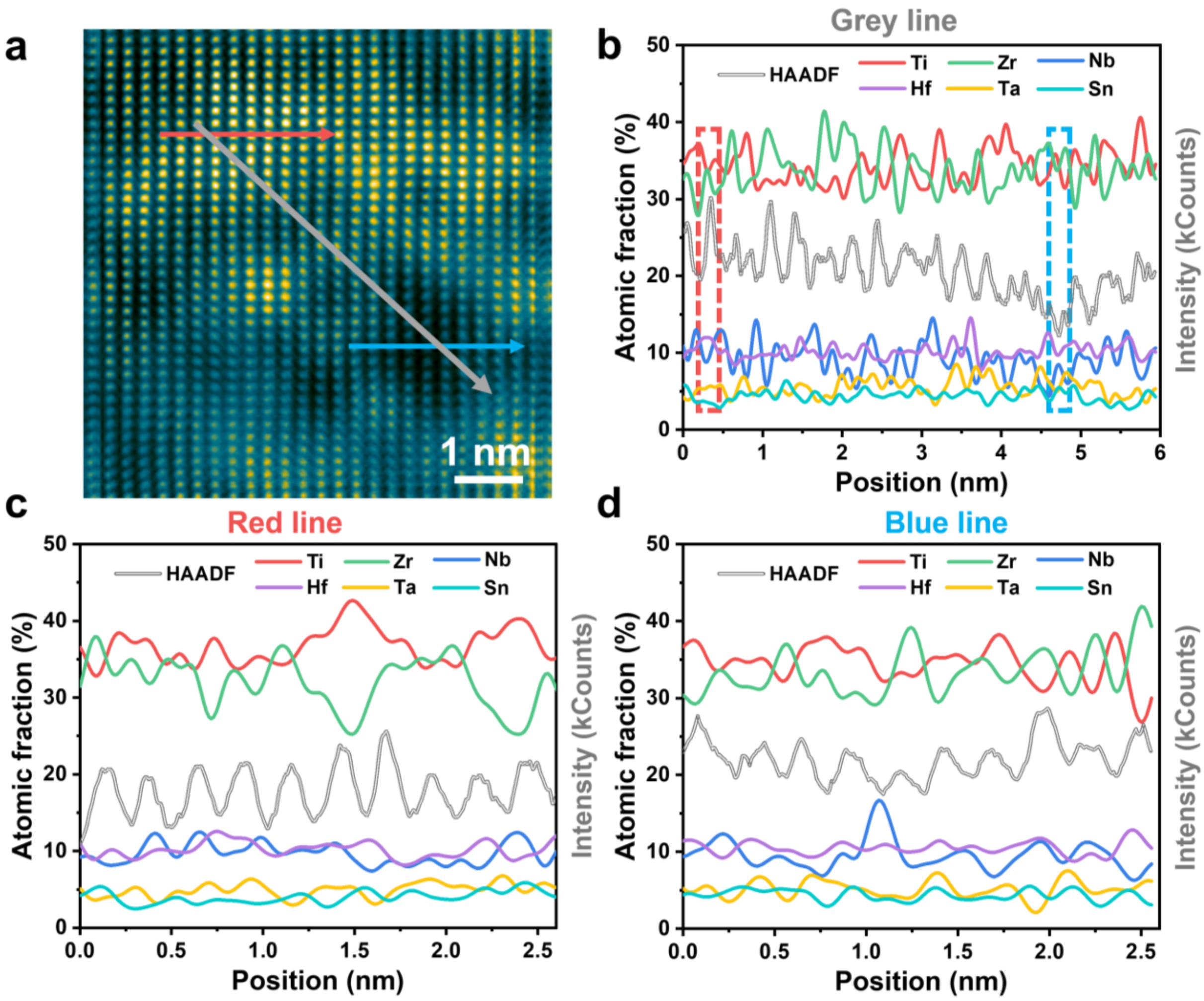

Supplementary Figure 6 | Atomic-scale chemical fluctuations in the $Hf_{10}Ta_5Sn_5$ alloy. **a**, Inverse fast Fourier transform image acquired from the region shown in Figure 2i, with arrows indicating the directions of elemental line scans across local chemical fluctuations. **b–d**, Elemental concentration profiles of Ti, Zr, Nb, Hf, Ta and Sn and the corresponding HAADF intensity profiles measured along the grey, red and blue arrows in **a**, respectively.

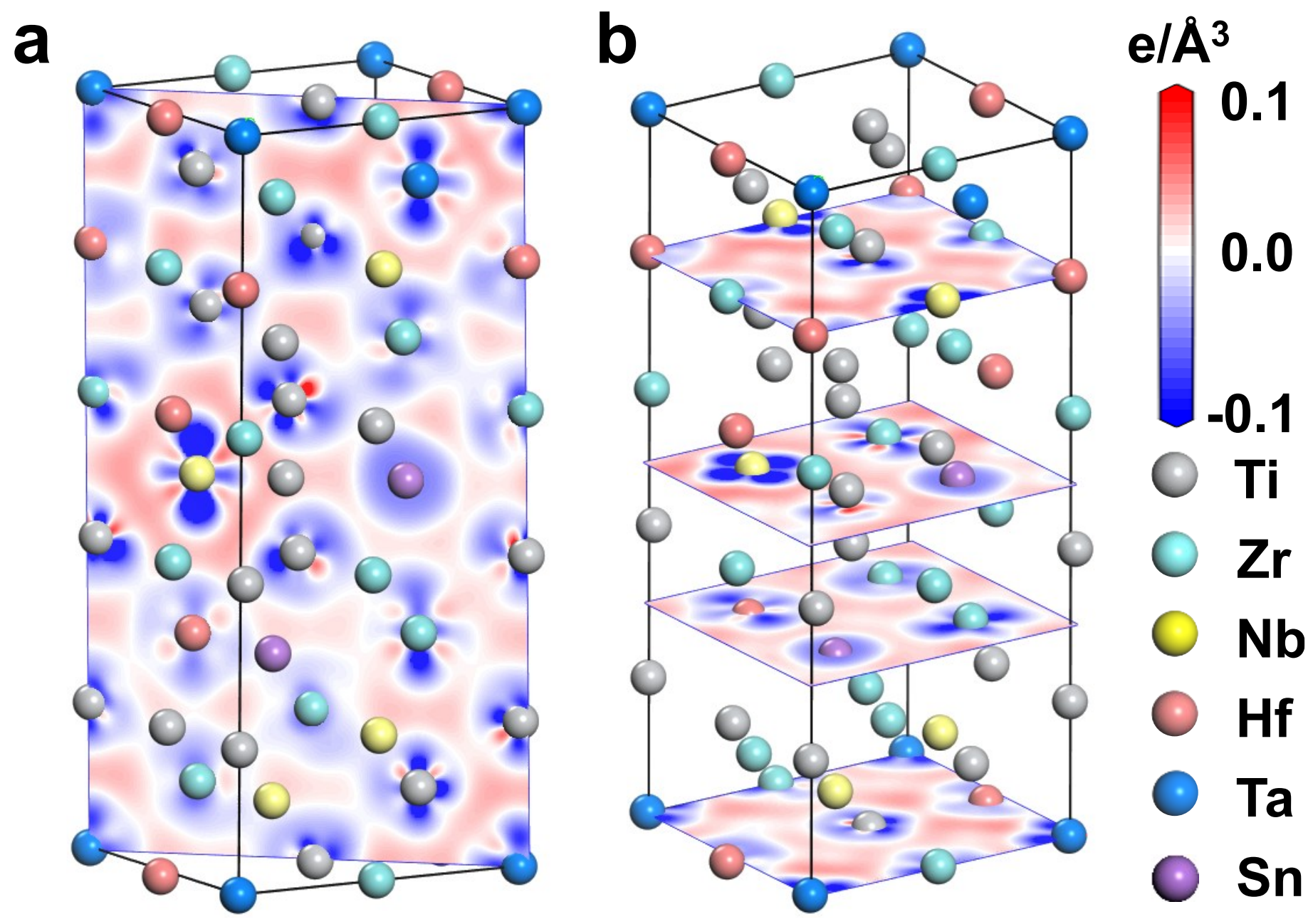

Supplementary Figure 7 | Charge-density difference maps and slice positions in the $Hf_{10}Ta_5Sn_5$ alloy. **a**, Charge-density difference map on the (110) plane used for the analysis in Figure 3e. **b**, Representative slice positions on the (100) planes used for the charge-density difference analysis in Figure 3f. Red and blue regions denote electron accumulation and depletion, respectively.

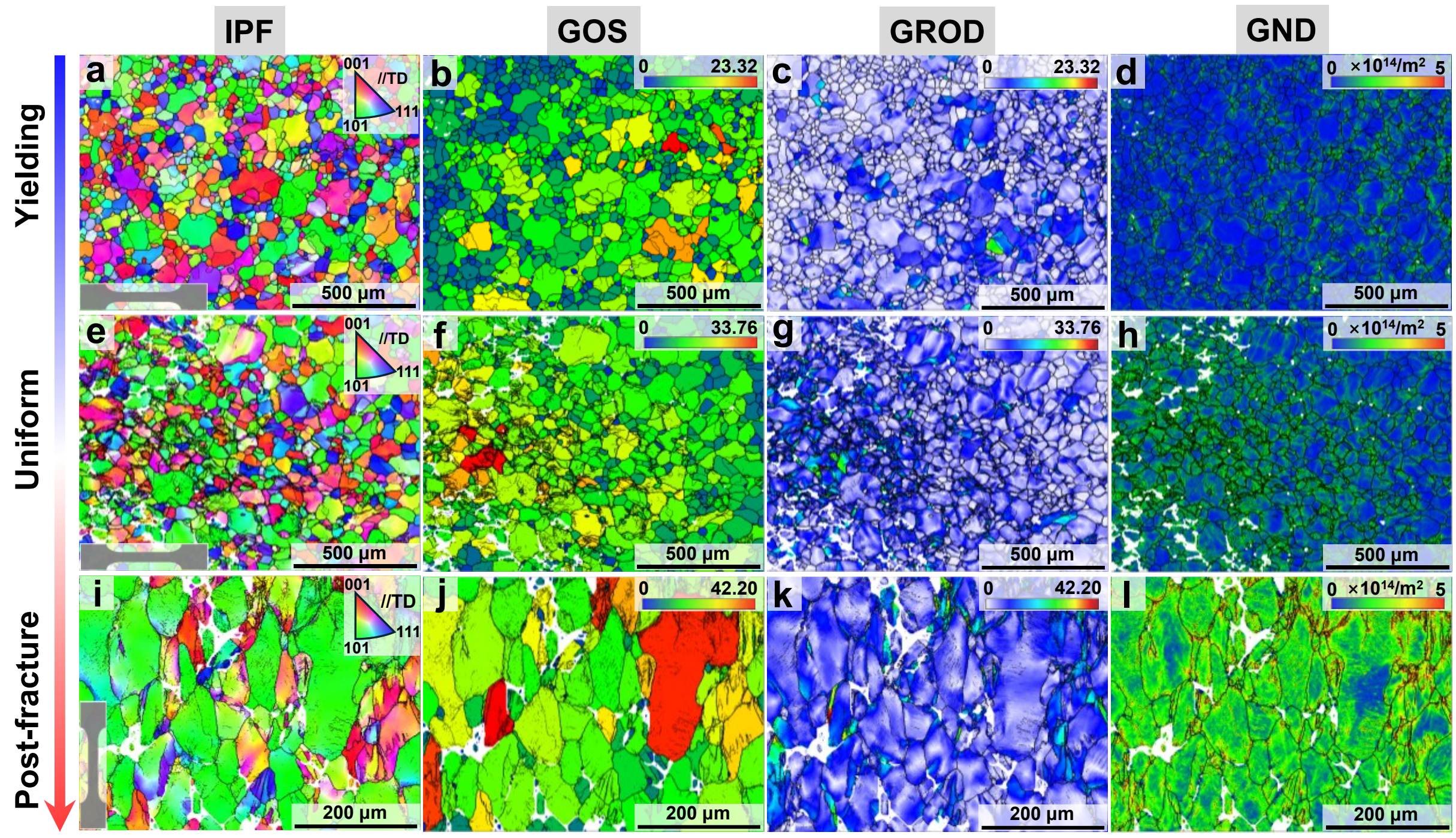

Supplementary Figure 8 | Evolution of EBSD microstructures in the $Hf_{10}Ta_5Sn_5$ alloy during tensile deformation. a–d, Electron backscatter diffraction (EBSD) inverse pole figure (IPF), grain orientation spread (GOS), grain reference orientation deviation (GROD) and geometrically necessary dislocation (GND) maps obtained at the yielding stage (~2% strain). **e–h**, Corresponding EBSD maps obtained during uniform deformation (~10% strain). **i–l**, Corresponding EBSD maps acquired from the post-fracture region. White regions denote unindexed pixels, which are mainly concentrated in strongly deformed areas where severe local lattice distortion degrades the EBSD pattern quality and prevents reliable indexing.

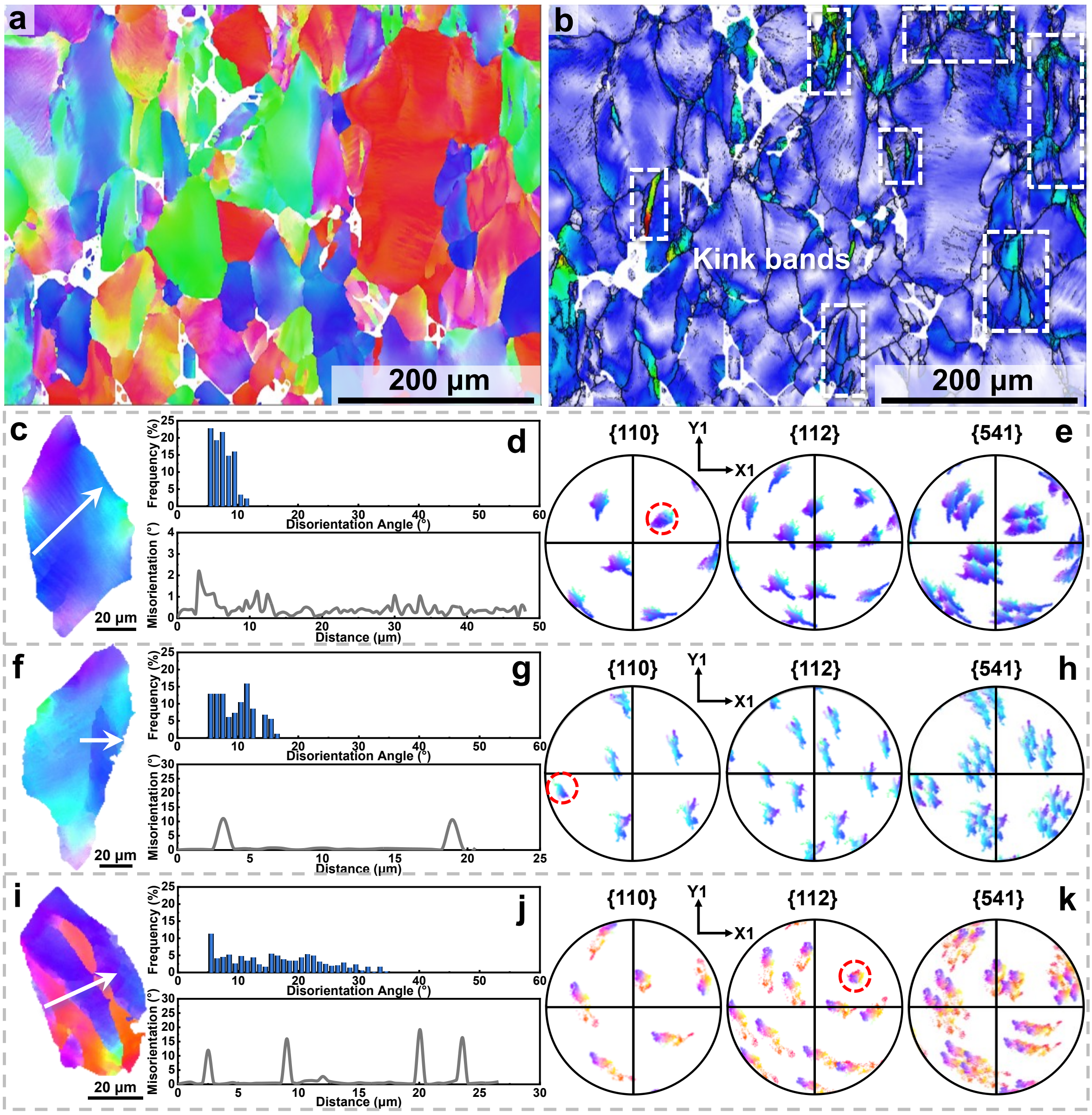

Supplementary Figure 9 | EBSD analysis of kink bands in the post-fracture $Hf_{10}Ta_5Sn_5$ alloy. a, b, IPF and GROD maps acquired from the post-fracture region, showing pronounced intragranular orientation gradients and banded deformation structures. White regions denote unindexed pixels associated with locally degraded EBSD pattern quality in strongly deformed areas. **c, f, i**, Representative banded regions selected from **b**, showing different degrees of kink-band development after fracture. The region in **c** retains low-angle parallel bands with relatively small point-to-point misorientation, whereas the regions in **f** and **i** exhibit sharper orientation gradients and larger localized misorientation peaks, characteristic of well-developed kink bands. **d, g, j**, Disorientation-angle distributions and point-to-point misorientation profiles measured along the arrows in **c**, **f** and **i**, respectively. **e, h, k**, Corresponding {110}, {112} and {541} pole figures, showing pole clustering mainly around <110> or <112> axes. Together with Figure 4, these post-fracture observations indicate further development of kink-band-related lattice rotation with increasing strain.

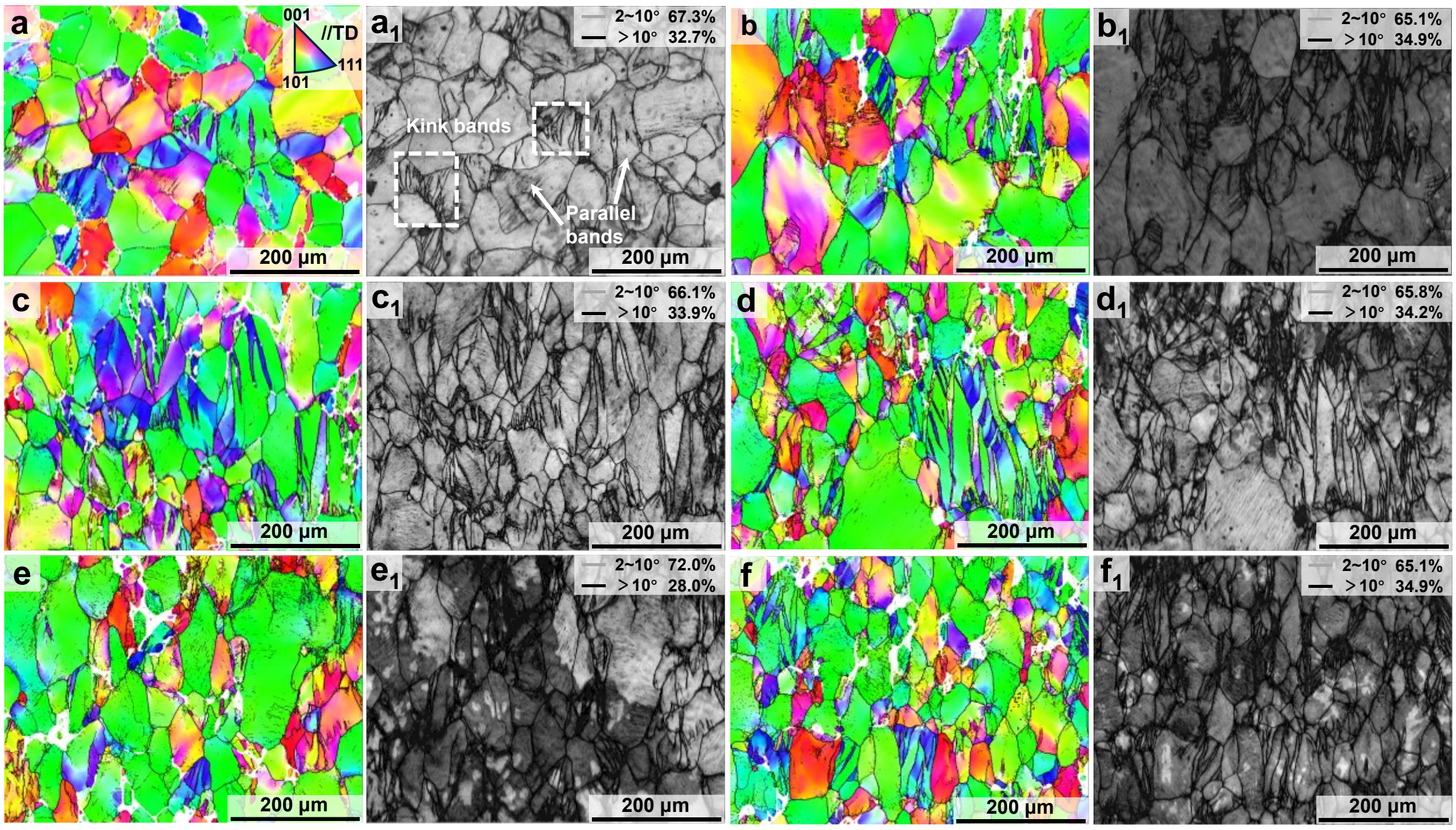

Supplementary Figure 10 | Post-fracture EBSD microstructures of the Ti–Zr–Nb–Hf–Ta(–Sn) alloys. **a–f**, IPF maps of the fractured $Hf_{10}Ta_{10}$, $Hf_{7.5}Ta_{10}Sn_{2.5}$, $Hf_5Ta_{10}Sn_5$, $Hf_{10}Ta_{7.5}Sn_{2.5}$, $Hf_{10}Ta_5Sn_5$ and $Hf_{7.5}Ta_{7.5}Sn_5$ alloys, respectively. White regions denote unindexed pixels associated with locally degraded EBSD pattern quality. **a_1–f_1**, Corresponding band-contrast maps with boundary overlays, revealing band-like deformation features and spatially heterogeneous diffraction-pattern quality. Dark regions indicate reduced band contrast, which is more pronounced in strongly deformed areas with substantial local lattice distortion. The indicated fractions of 2–10° and >10° boundaries describe the local boundary/misorientation structures within the mapped regions. These maps are used as representative qualitative evidence of kink-band-related deformation across the alloy series, rather than as a quantitative measure of kink-band density. TD denotes the tensile direction.

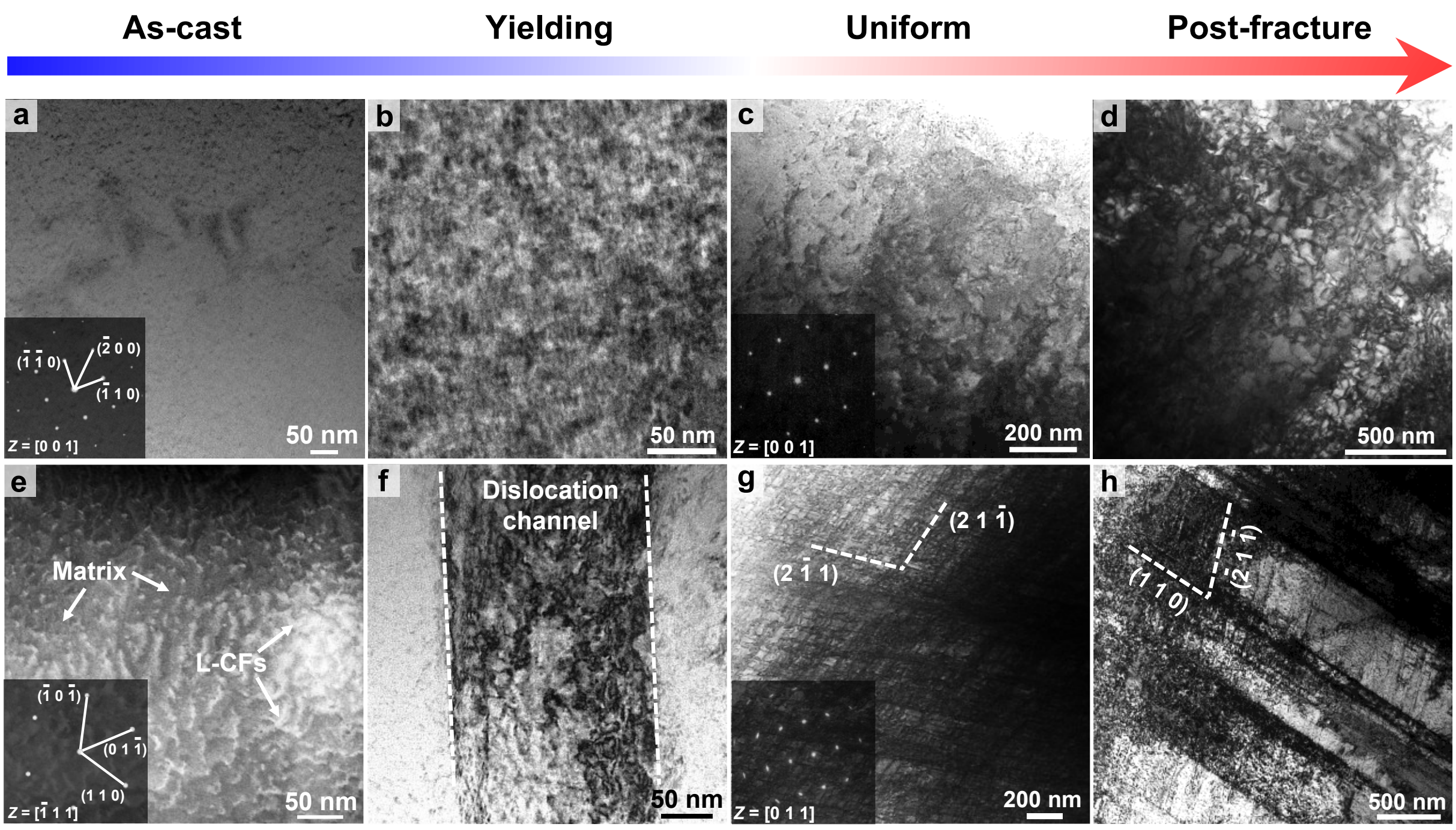

Supplementary Figure 11 | Comparative evolution of TEM deformation microstructures in the $Hf_{10}Ta_{10}$ and $Hf_{10}Ta_5Sn_5$ alloys. a–d, TEM images of the $Hf_{10}Ta_{10}$ alloy in the as-cast, yielding, uniform deformation and post-fracture states, respectively. **e–h**, Corresponding TEM images of the $Hf_{10}Ta_5Sn_5$ alloy, showing long-range chemical fluctuations in the as-cast state, localized dislocation-channel formation at yielding, and kink-band-mediated deformation during uniform deformation and after fracture. The diffraction inset in **g** shows rotated diffraction spots, indicating lattice rotation associated with kink-band formation during uniform deformation.

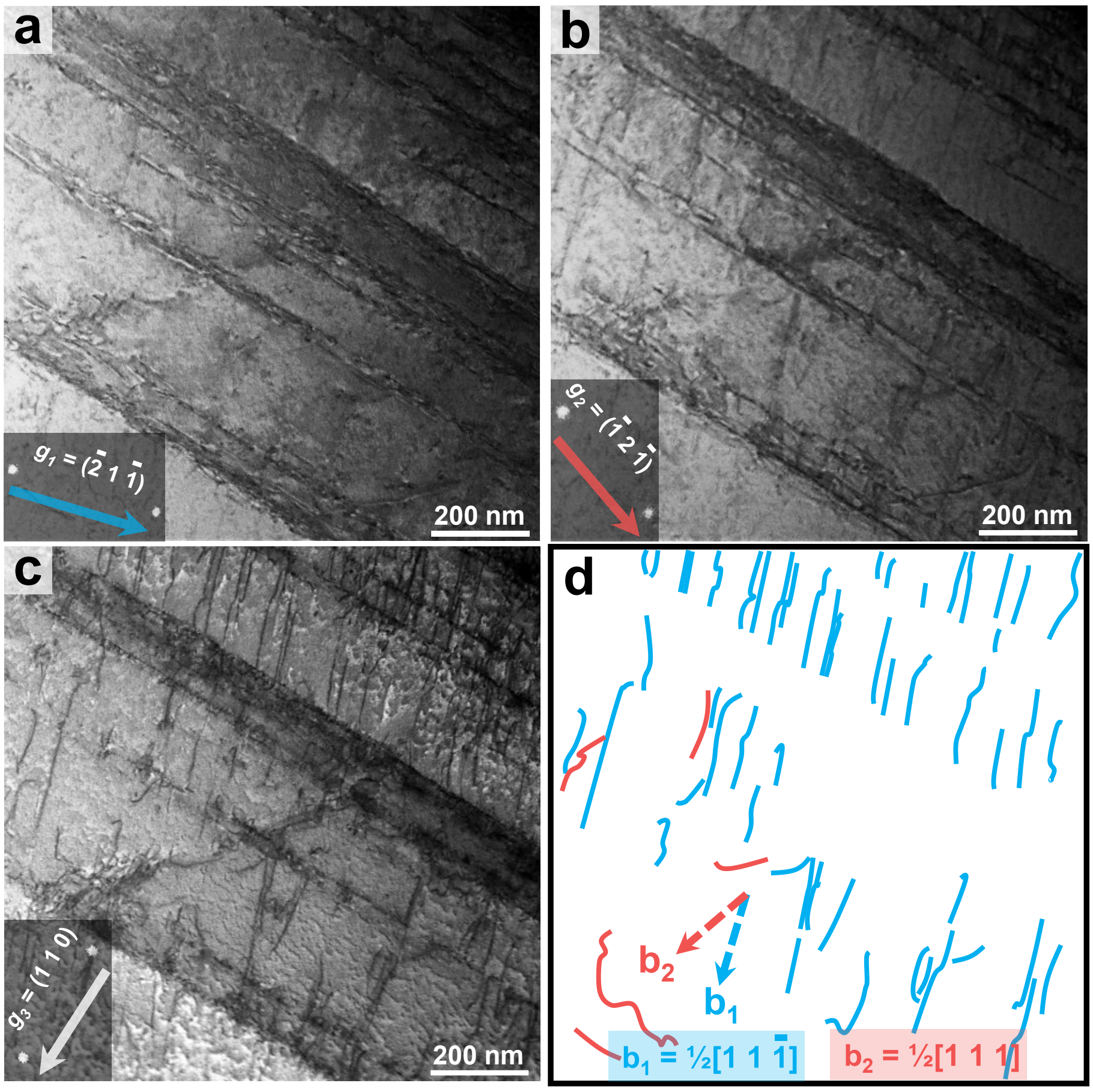

Supplementary Figure 12 | Two-beam TEM analysis of dislocations in the fractured $Hf_{10}Ta_5Sn_5$ alloy. a–c, Bright-field TEM images acquired under two-beam conditions using diffraction vectors of $\boldsymbol{g}_1 = (\bar{2}1\bar{1})$, $\boldsymbol{g}_2 = (\bar{1}2\bar{1})$ and $\boldsymbol{g}_3 = (110)$, respectively. **d**, Schematic of the visible dislocations and Burgers-vector assignments based on $\boldsymbol{g} \cdot \boldsymbol{b}$ invisibility analysis.

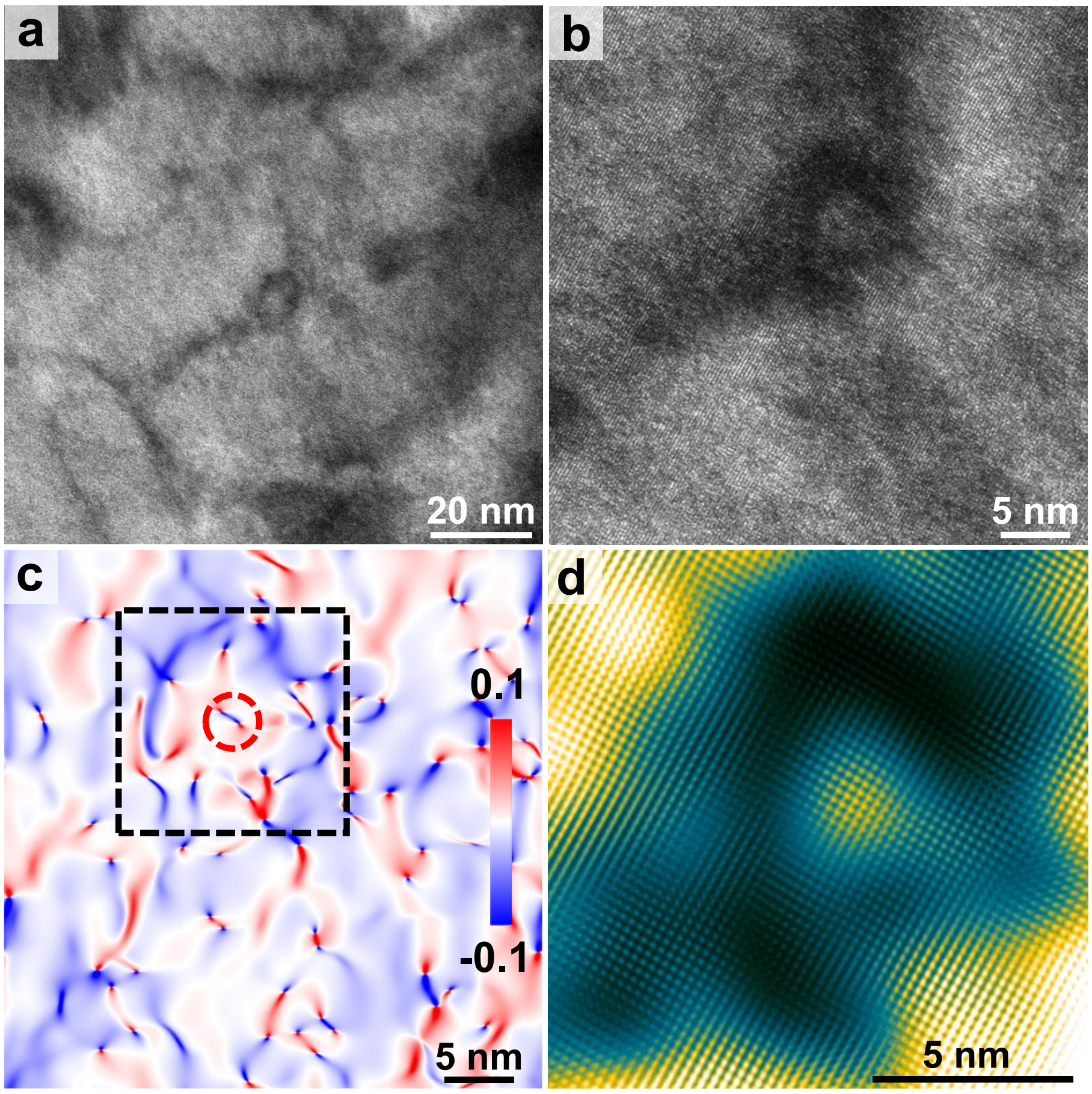

Supplementary Figure 13 | Local interaction between a dislocation and nanoscale fluctuation-associated strain heterogeneity in the deformed $Hf_{10}Ta_5Sn_5$ alloy. a, TEM image showing a locally pinned dislocation configuration. **b**, HRTEM image of the interaction region. **c**, Corresponding GPA strain map showing pronounced local strain heterogeneity around the dislocation interaction site. **d**, IFFT reconstruction highlighting nanoscale fluctuation-associated lattice contrast in the corresponding region.

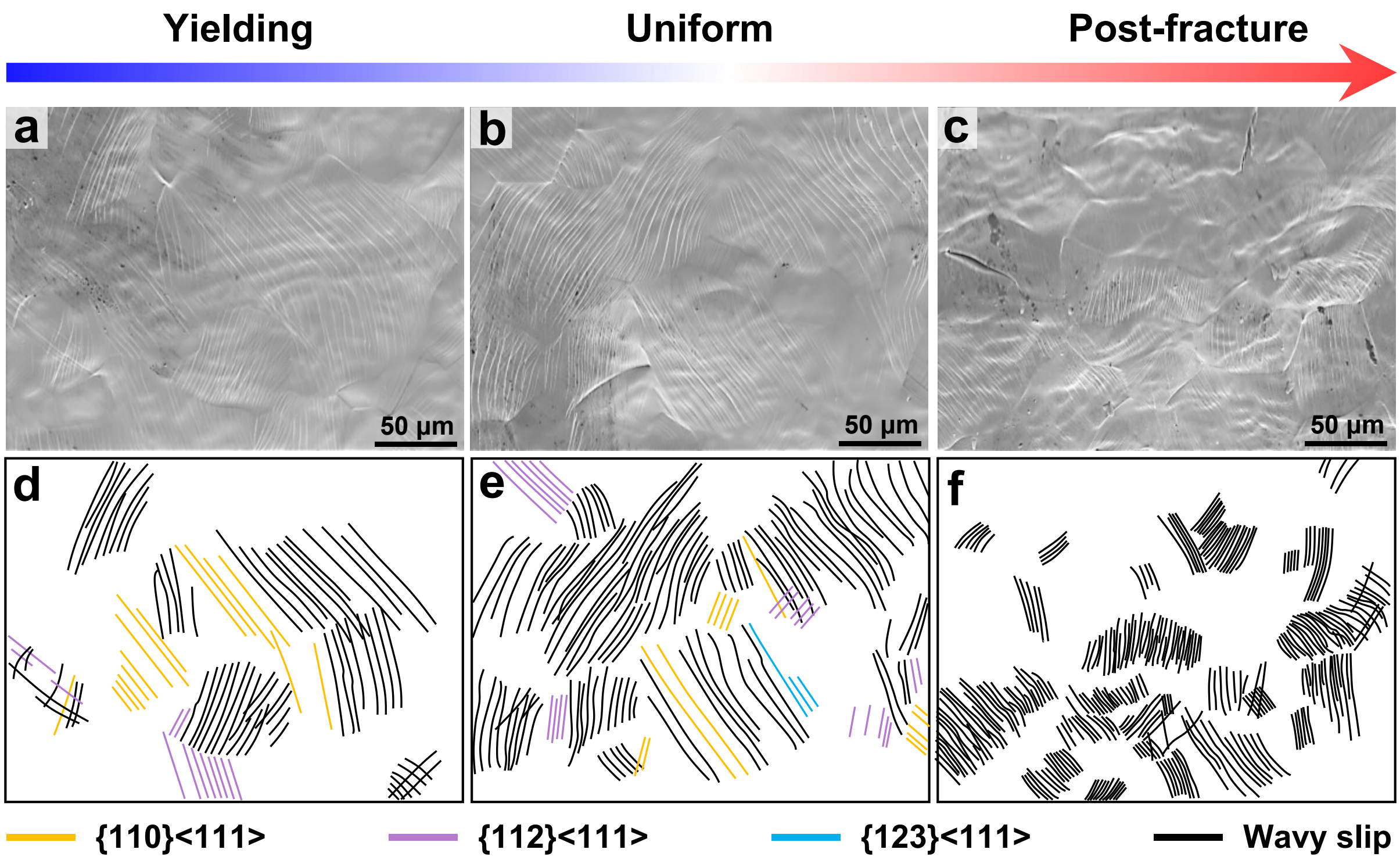

Supplementary Figure 14 | Stage-dependent slip-trace evolution in the $Hf_{10}Ta_5Sn_5$ alloy. a–c, Surface slip morphologies at the yielding stage (~2% strain), uniform deformation stage (~10% strain) and post-fracture state, respectively. The fracture strain is approximately 20%. **d–f**, Corresponding slip-trace analysis showing wavy-slip features and the activation of {110}<111>, {112}<111> and {123}<111> slip systems.

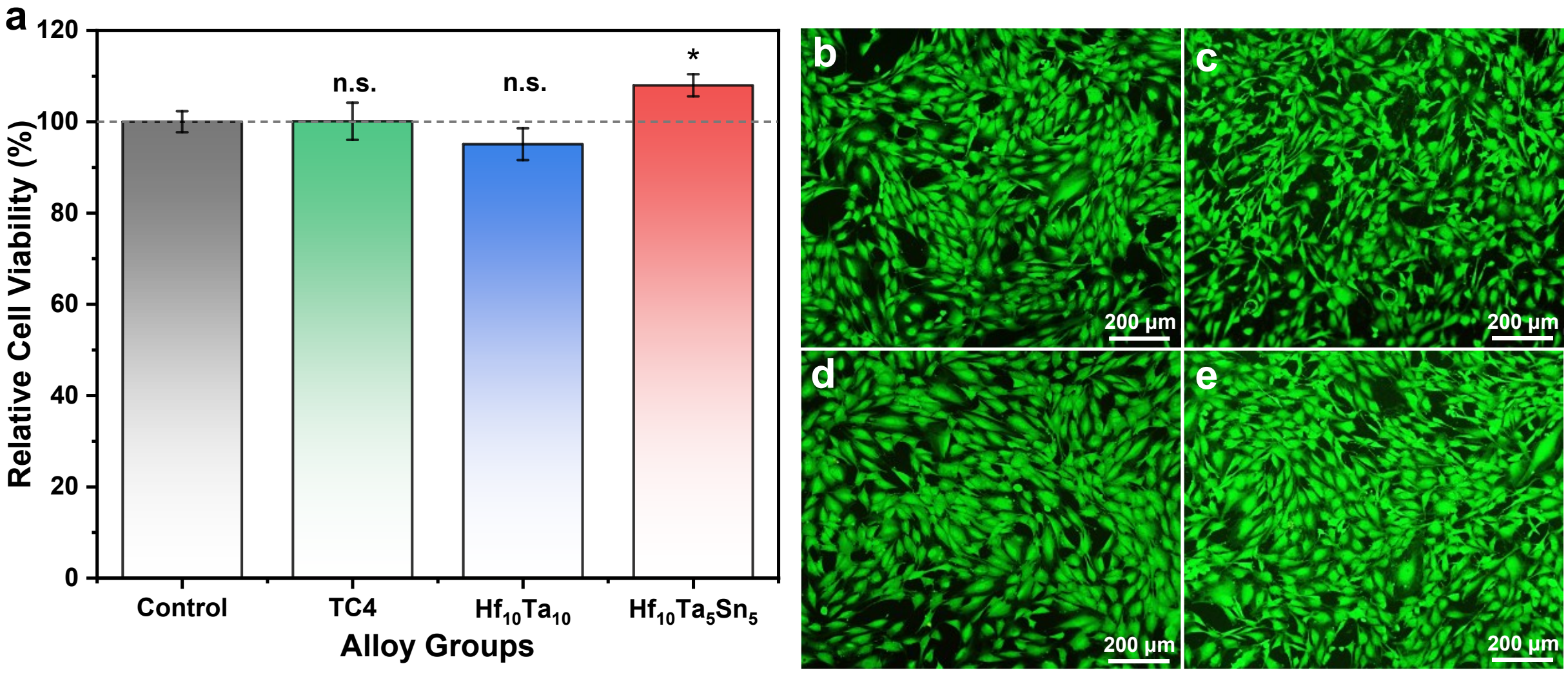

Supplementary Figure 15 | Cytocompatibility evaluation of the $Hf_{10}Ta_{10}$ and $Hf_{10}Ta_5Sn_5$ alloys. a, Relative cell viability of MC3T3-E1 cells cultured on TC4, $Hf_{10}Ta_{10}$ and $Hf_{10}Ta_5Sn_5$ specimens for 72 h, with cells cultured without alloy specimens used as the control group. The dashed line indicates the viability level of the control group. The $Hf_{10}Ta_5Sn_5$ alloy shows a relative cell viability of 108.0 ± 2.4%, higher than those of TC4 (100.1 ± 4.1%) and $Hf_{10}Ta_{10}$ (95.1 ± 3.5%) in the same assay. **b–e**, Representative fluorescence-staining images of cells cultured in the control, TC4, $Hf_{10}Ta_{10}$ and $Hf_{10}Ta_5Sn_5$ groups, respectively. Data are presented as mean ± s.d.; n.s., not significant; *$P < 0.05$ versus the control group.

Supplementary Table 1 | Elemental physical and d-electron parameters used for alloy-design calculations[6, 10, 11, 12]**.**

Elemental parameter	Ti	Zr	Nb	Hf	Ta	Sn
T_m (K)	1941	2128	2750	2506	3290	505
r (nm)	0.1430	0.1551	0.1429	0.1541	0.1430	0.1620
E (GPa)	116	88	105	78	186	50
G (GPa)	44	33	38	30	69	18
Bo	2.790	3.086	3.099	3.110	3.144	2.283
Md (eV)	2.447	2.934	2.424	2.975	2.531	2.100

Supplementary Table 2 | Alloy-design parameters and tensile properties of the designed Sn-containing alloys and the Sn-free parent reference.

Alloys	VEC	$\overline{Bo}$	$\overline{Md}$ (eV)	ΔH_{mix} (kJ/mol)	ΔS_{mix} (J/(mol·K))	Young's modulus E (GPa)	Yield strength σ_y (MPa)	Fracture elongation δ (%)
$Hf_{10}Ta_{10}$	4.20	2.992	2.676	1.68	13.38	54.6 ± 0.4	975 ± 14	25.6 ± 0.5
$Hf_{7.5}Ta_{10}Sn_{2.5}$	4.20	2.971	2.654	-0.93	12.32	49.4 ± 0.6	775 ± 18	18.1 ± 0.7
$Hf_5Ta_{10}Sn_5$	4.20	2.951	2.633	-3.37	12.43	53.7 ± 0.5	1238 ± 21	13.8 ± 0.6
$Hf_{10}Ta_{7.5}Sn_{2.5}$	4.18	2.970	2.666	-1.11	12.32	47.1 ± 0.7	715 ± 16	18.1 ± 0.8
**$Hf_{10}Ta_5Sn_5$**	**4.15**	**2.949**	**2.655**	**-3.89**	**12.43**	**52.8 ± 0.4**	**1143 ± 15**	**20.3 ± 0.5**
$Hf_{7.5}Ta_{7.5}Sn_5$	4.18	2.950	2.644	-3.62	12.50	50.8 ± 0.5	950 ± 19	14.6 ± 0.7

Supplementary Table 3 | STEM-EDS compositions of L-CFs and the surrounding β matrix in the $Hf_{10}Ta_5Sn_5$ alloy (at.%).

Alloy	Region	Ti	Zr	Nb	Hf	Ta	Sn
$Hf_{10}Ta_5Sn_5$	L-CF (bright region)	32.87	33.95	9.57	11.03	6.26	6.32
	β Matrix (dark region)	34.63	34.86	10.47	9.78	4.97	5.29

Supplementary References

[1] J. Wang, L. Zhu, M. Zhou, L. Kong, J. Li, $Ti_{35}Zr_{35}Nb_{10}Hf_{20-x}Ta_x$ high-entropy alloys with exceptional performance as biomedical load-bearing materials, *Journal of Materials Science & Technology* **2026**, *241*, 298, https://doi.org/10.1016/j.jmst.2025.02.096.

[2] J. Wang, M. Zhou, L. Zhu, et al., Synergistic effects of Hf and Ta on strength-ductility in refractory high-entropy alloys: Kink band formation and dislocation evolution, *Acta Materialia* **2025**, *300*, 121492, https://doi.org/10.1016/j.actamat.2025.121492.

[3] A. Takeuchi, A. Inoue, Classification of bulk metallic glasses by atomic size difference, heat of mixing and period of constituent elements and its application to characterization of the main alloying element, *Materials Transactions* **2005**, *46* (12), 2817, https://doi.org/10.2320/matertrans.46.2817.

[4] C.-T. Wu, H.-T. Chang, C.-Y. Wu, et al., Machine learning recommends affordable new Ti alloy with bone-like modulus, *Materials Today* **2020**, *34*, 41, https://doi.org/10.1016/j.mattod.2019.08.008.

[5] A. Biesiekierski, J. Wang, M. A. Gepreel, C. Wen, A new look at biomedical Ti-based shape memory alloys, *Acta Biomaterialia* **2012**, *8* (5), 1661, https://doi.org/10.1016/j.actbio.2012.01.018.

[6] M. Abdel-Hady, K. Hinoshita, M. Morinaga, General approach to phase stability and elastic properties of β-type Ti-alloys using electronic parameters, *Scripta Materialia* **2006**, *55* (5), 477, https://doi.org/10.1016/j.scriptamat.2006.04.022.

[7] Q. J. Li, H. Sheng, E. Ma, Strengthening in multi-principal element alloys with local-chemical-order roughened dislocation pathways, *Nature Communications* **2019**, *10* (1), 3563, https://doi.org/10.1038/s41467-019-11464-7.

[8] Y. Lu, Y. H. Zhang, E. Ma, W. Z. Han, Relative mobility of screw versus edge dislocations controls the ductile-to-brittle transition in metals, *Proceedings of the National Academy of Sciences of the United States of America* **2021**, *118* (37), e2110596118, https://doi.org/10.1073/pnas.2110596118.

[9] Y. B. Chun, M. Battaini, C. H. J. Davies, S. K. Hwang, Distribution characteristics of in-grain misorientation axes in cold-rolled commercially pure titanium and their correlation with active slip modes, *Metallurgical and Materials Transactions A: Physical Metallurgy and Materials Science* **2010**, *41* (13), 3473, https://doi.org/10.1007/s11661-010-0410-4.

[10] T. Li, S. Wang, W. Fan, et al., CALPHAD-aided design for superior thermal stability and mechanical behavior in a TiZrHfNb refractory high-entropy alloy, *Acta Materialia* **2023**, *246*, 118728, https://doi.org/10.1016/j.actamat.2023.118728.

[11] O. N. Senkov, J. M. Scott, S. V. Senkova, D. B. Miracle, C. F. Woodward,

Microstructure and room temperature properties of a high-entropy TaNbHfZrTi alloy, *Journal of Alloys and Compounds* **2011**, *509* (20), 6043, https://doi.org/10.1016/j.jallcom.2011.02.171.

[12] D. B. Miracle, O. N. Senkov, A critical review of high entropy alloys and related concepts, *Acta Materialia* **2017**, *122*, 448, https://doi.org/10.1016/j.actamat.2016.08.081.
